# New bobtail squid (Sepiolidae: Sepiolinae) from the Ryukyu islands revealed by molecular and morphological analysis

**DOI:** 10.1038/s42003-019-0661-6

**Published:** 2019-12-11

**Authors:** Gustavo Sanchez, Jeffrey Jolly, Amanda Reid, Chikatoshi Sugimoto, Chika Azama, Ferdinand Marlétaz, Oleg Simakov, Daniel S. Rokhsar

**Affiliations:** 1 Molecular Genetics Unit, Okinawa Institute of Science and Technology Graduate University, Onna, Okinawa 904-0495 Japan; 2 Graduate School of Integrated Sciences for Life, Hiroshima University, Higashi Hiroshima, Hiroshima, Japan; 3 Australian Museum Research Institute, 1 William Street, Sydney, Australia 2010; 4 Department of Molecular Evolution and Development, University of Vienna, Vienna, Austria; 5 Department of Molecular and Cell Biology, Life Sciences Addition #3200, Berkeley, CA 94720-3200 USA

**Keywords:** Evolution, Ecology, Molecular ecology, Ecology, Molecular ecology

## Abstract

Bobtail squid are emerging models for host–microbe interactions, behavior, and development, yet their species diversity and distribution remain poorly characterized. Here, we combine mitochondrial and transcriptome sequences with morphological analysis to describe three species of bobtail squid (Sepiolidae: Sepiolinae) from the Ryukyu archipelago, and compare them with related taxa. One Ryukyuan type was previously unknown, and is described here as *Euprymna brenneri* sp. nov. Another Ryukyuan type is morphologically indistinguishable from *Sepiola parva* Sasaki, 1913. Molecular analyses, however, place this taxon within the genus *Euprymna* Steenstrup, 1887, and additional morphological investigation led to formal rediagnosis of *Euprymna* and reassignment of this species as *Euprymna parva* comb. nov. While no adults from the third Ryukyuan type were found, sequences from hatchlings suggest a close relationship with *E. pardalota* Reid, 2011, known from Australia and East Timor. The broadly sampled transcriptomes reported here provide a foundation for future phylogenetic and comparative studies.

## Abstract (web summary)

Gustavo Sanchez et al. describe three species of bobtail squid from the Ryukyu archipelago using morphological analysis, mitochondrial sequences, and transcriptome sequences. One of these species was a previously unknown bobtail squid from the genus *Euprymna*, the second is reassigned to the genus *Euprymna*, and the third species is closely related to another bobtail squid endemic from Australia and East Timor.

## Introduction

Bobtail squid of the subfamily Sepiolinae Appellöf, 1898, including *Euprymna* Steenstrup, 1887 and *Sepiola* Leach, 1817, are small nektobenthic cephalopods generally found in shallow coastal waters of the Indo-west Pacific, the east Atlantic coast, and the Mediterranean Sea. The common name of these animals comes from their characteristic rounded (“bobbed”) posterior mantle. Bobtail squid lack a “true” sepion (cuttlebone) and the gladius (pen) is chitinous, reduced, or absent. Together with Idiosepiidae they are the sister group of the true squids (Teuthoidea)^1^.

Due to their small size and ease of culturing in captivity, species of *Euprymna* and *Sepiola* are increasingly emerging as model systems for a range of biological studies^2–4^. The Hawaiian bobtail squid *Euprymna scolopes* Berry, 1913 has become a prominent model for the study of symbiosis with their luminescent bacteria *Vibrio fischeri* located in the luminescent organ^5,6^. Associative learning, behavioral genetic studies, and the heritability of personality and fitness traits were also investigated in several species of bobtail squids^7–11^. The cultivation of *E. scolopes*, initially for studies of symbiosis, has promoted the detailed characterization of embryonic development, both morphologically^12^ and at the molecular level^13–15^. The small size of bobtail squid makes them ideal for advanced imaging^16^ opening up new opportunities for characterizing the cephalopod nervous system, complementing the extensive studies of the pygmy squid *Idiosepius* Steenstrup, 1881 by Shigeno and colleagues^17–19^. With the closure of the life cycle for several sepiolid squid^20–23^ (including *Euprymna parva* (Sasaki, 1913) comb. nov. described below), and the recently sequenced genome of *E. scolopes*^24^, these small cephalopods are emerging systems for comparative biology.

Over 68 species of Sepiolidae have been described^25^. Among the Sepiolinae, species of *Euprymna* can be distinguished by the absence of a distinct copulatory apparatus in their hectocotylus, the modified left arm 1 in males involved in mating, and the number and organization of arm sucker rows. Within *Euprymna*, in addition to the differences in the hectocotylus among species, the nature and arrangement of male enlarged arm suckers are among the key characters used to distinguish species. Unfortunately, the suckers readily become dislodged from preserved specimens during examination, often confounding definitive taxonomic identification. As in all Sepiolinae, most distinguishing morphological characters are found only in males, making it very difficult to identify females using morphology alone. The potential for phenotypic plasticity as described in males of *Sepiola birostrata* Sasaki, 1918^26^ and the shortage of distinguishing morphological characters for species of the Indo-Pacific *Euprymna* and *Sepiola* are also problematic.

More than a century of classical morphology-based taxonomy has more recently been augmented by surveys of the mitochondrial cytochrome oxidase I (COI) marker in bobtail squids^27–36^, especially in the subfamily Sepiolinae. While COI molecular characters (i.e., variable sites) supplement morphological features in this taxonomically tricky group of cephalopods, several misidentifications have been reported for the North Sea *Sepiola* species after COI sequencing and morphological analysis^37^. Transcriptome sequences complement DNA barcoding and morphological analysis by providing sequence data from multiple unlinked nuclear coding regions. Information from these loci enables more accurate species identification and the reconstruction of their phylogenetic relationships.

Here, we characterize bobtail squid from the Okinawa and Yaeyama islands of the Ryukyu archipelago, a region known for its rich marine diversity^38^. Our aim is to describe the sepiolines of the Ryukyus and set them within the broader systematic framework of Indo-Pacific bobtail squid. We found three different species of bobtail squid in these waters, and characterized them using COI sequences, transcriptome sequences, and morphology. One Ryukyuan species, recognized by the molecular traits of laboratory hatchlings, appears to be closely related to *Euprymna pardalota* Reid 2011 described from northern Australia. A second species is indistinguishable from *Sepiola parva* Sasaki 1913, but comparative molecular analysis and reexamination of morphology indicates that this taxon should instead be assigned to the genus *Euprymna*, which we also revise. The third Ryukyuan species is morphologically and molecularly distinct from other sepiolines. We formally describe this new species here and name it in honor of Sydney Brenner, pioneering molecular geneticist and supporter of Okinawan science.

This study is the most comprehensive COI analysis of the Sepiolidae published to date. The optic lobe transcriptome sequences reported here will prove valuable for resolving the phylogenetic relationships among this increasingly studied group of cephalopods.

## Results

**Fig. 1:**
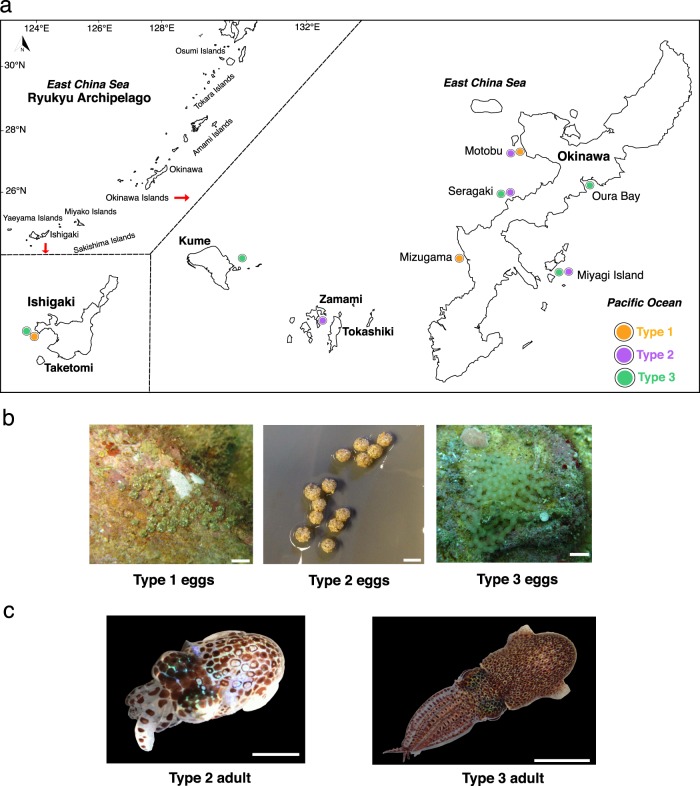
Ryukyuan bobtail collection sites, eggs masses, and adult morphotypes. **a** Ryukyu Archipelago collection sites. **b** Ryukyuan bobtail squid egg clutches from Types 1–3; scale bars 5, 4 and 12 mm, respectively. **c** Live adult Type 2 and Type 3 collected during surveys, scale bars 5 and 11 mm, respectively.

**Fig. 2:**
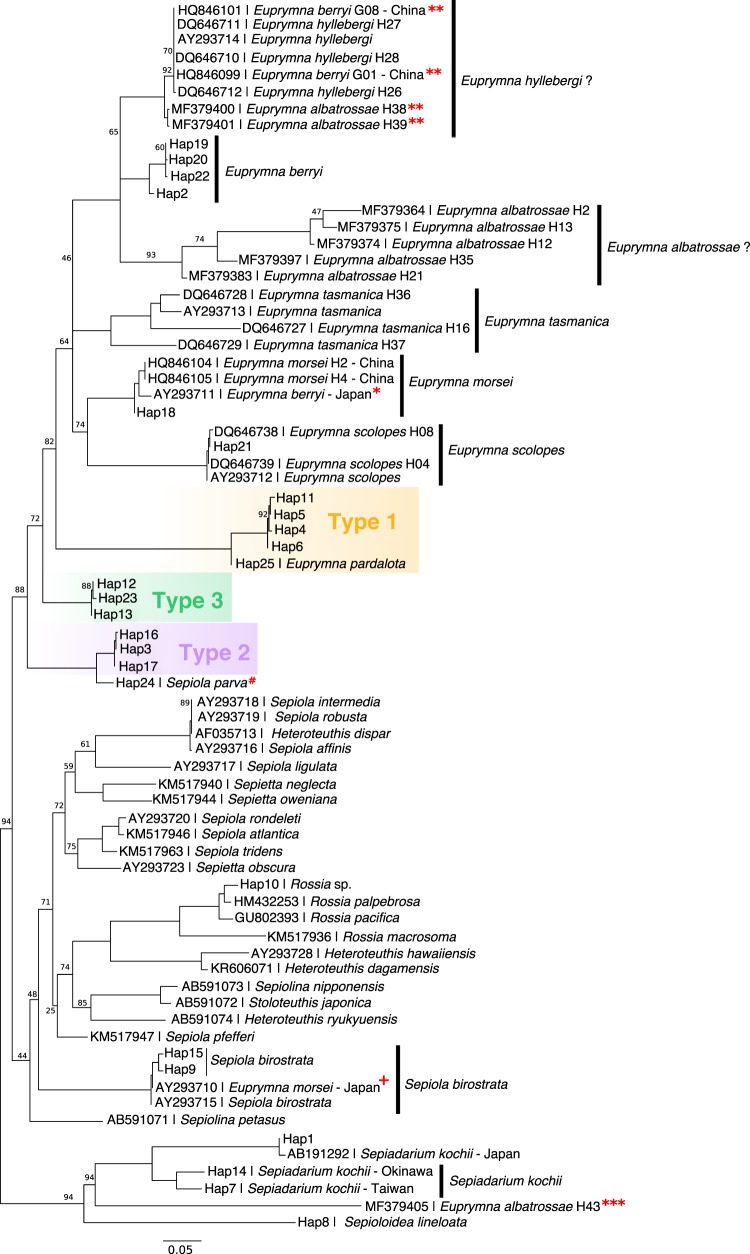
Maximum likelihood tree estimated for COI sequences. Possible misidentifications of original taxa: *suspected *E. morsei* (Verrill, 1881); **suspected *E. hyllebergi* Nateewathana, 1997; ***suspected misidentification, likely sepiadariid; Sepiadariidae; *+*, likely *S. birostrata* (see Supplementary Table 1). Nodes with bootstrap support <95% are shown. ^**#**^We have reassigned this species to *Euprymna* below.

We found eggs of three distinct sizes and/or clutch morphologies while collecting around the Ryukyu islands (Fig. 1a), and designated them Types 1–3 (Fig. 1b) (see Materials and methods). We consistently obtained hatchlings for all three egg types in laboratory aquaria (Table 1, Methods), enabling genetic analysis and sequence comparison with adults. We found only two distinct adult types (Fig. 1c) and associated these adult forms with Types 2 and 3 hatchlings using mitochondrial COI sequences (Fig. 2). Types 2 and 3 hatchlings could be raised to reproductively mature adults in the laboratory (Methods), further corroborating their match with the two adult types found in the wild. During our samplings from February to November over several years (Methods) we did not find any Type 1 adults, and Type 1 hatchlings were recalcitrant to culture.

**Table 1:** Characteristics of Ryukyuan bobtail eggs and hatchlings.

Trait	Type 1	Type 2	Type 3
Egg size + jelly coat + sand	2.5 mm	4 mm	4 mm
Egg size (minus jelly coat)	1.5 mm	2 mm	3 mm
Egg clustering pattern	Individually laid	Individually laid	Laid as a cluster
Typical number of eggs found per clutch	>50	13–20	>100
Number of embryos found per egg unit	1	1 or 2 (for eggs laid in captivity)	1
Dorsal mantle length at hatching	1.5 mm	1.5 mm	2 mm
Swimming behavior at hatching	Swimming near surface	Benthic	Swimming near surface
Dorsal Mantle Length at maturity	Unknown	12–15 mm	30–40 mm
Number of rows of suckers	Two	Two	Four
Color of hatchlings	Dark brown	Dark brown	Dark brown
Positive phototaxic	Yes	Not obvious	Actively swimming towards light source
Food at hatching	Unsuccessful	Juvenile mysids (2–4 mm)	Adult mysids (5–10 mm)
Hunting pattern at hatching	Unsuccessful	Uses tentacles to seize prey while striking from below	Jets forward and seizes prey from below; holds prey with arms while eating

### COI phylogeny

To examine relationships among the Ryukyuan types and other species, we used maximum likelihood to estimate a phylogenetic tree with haplotypes from COI sequences (Fig. 2), combining publicly deposited data (Supplementary Table 1) with 41 additional sequences from our Ryukyuan samples and 42 new sequences from 9 previously described bobtail and related squid species (Materials and methods; Supplementary Table 2). We found that Ryukyuan Type 1 formed a clade with, and is clearly related to, *E. pardalota*, a species described from northern Australia^39^. Ryukyuan Type 2 lies within a clade identified as *Sepiola parva* Sasaki, 1913 from Sagami Bay in mainland Japan, near the type locality for this species. The *Sepiola parva* clade (including the Ryukyuan Type 2), however, lies within the *Euprymna* clade, and appears to render the genus *Euprymna* as polyphyletic. We confirm this placement below with both transcriptome data and morphological analysis, and propose a revised assignment of *Sepiola parva* to the genus *Euprymna*. The COI sequence for Ryukyuan Type 3 did not match any of the sequences deposited in Genbank to date.

### Transcriptome-based phylogeny

To avoid dependence on a single mitochondrial marker, for additional phylogenetic resolution, and to capture the nuclear variation within and between species, we also systematically sampled transcriptomes of hatchling and/or adult Ryukyuan bobtail squid types along with 17 individuals identified from six known Indo-Pacific sepiolines (Materials and methods; Supplementary Data). RNA from optic lobe was preferred because this organ is easily and reproducibly dissected, and provides deep coverage of genes associated with neural and other functions, although mantle tissue was also used for some individuals. While we were unable to obtain samples of *Euprymna hyllebergi*, reportedly found in the Gulf of Thailand, the Andaman Sea^23,40^ and the South China Sea^41^, this species is clearly distinct from our Ryukyuan types based on COI analyses (Fig. 2) and morphology. For outgroups, we added transcriptome data from the tropical bottletail squid *Sepiadarium kochii* Steenstrup, 1881, and the pyjama squid *Sepioloidea lineolata* (Quoy & Gaimard, 1832), both members of the family Sepiadariidae, which appears to be the sister taxon to the Sepiolidae^42–44^.

**Fig. 3:**
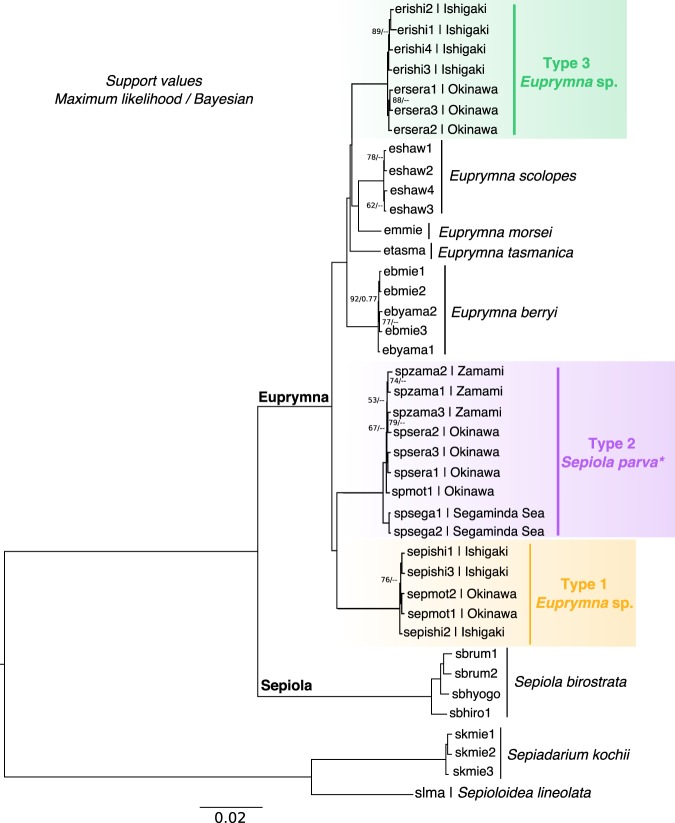
Maximum likelihood and Bayesian tree based on 452 orthologous genes using their coding sequences. Nodes with bootstrap support <95% and posterior probability <0.9 are shown. *Members of the Type 2 clade are morphologically indistinguishable from *Sepiola parva*. Based on the COI analyses, this phylogeny and our morphological analysis, however, we have redefined the genus *Euprymna* and reassigned this species to *Euprymna*.

Both maximum likelihood and Bayesian phylogenetic analyses obtained from 452 orthologous bobtail squid genes show that our three Ryukyuan types form three well-separated clades, consistent with their distinct egg mass types, mitochondrial COI, and (for Types 2 and 3) adult morphology (Fig. 3).

### Classification of Ryukyuan species

**Fig. 4:**
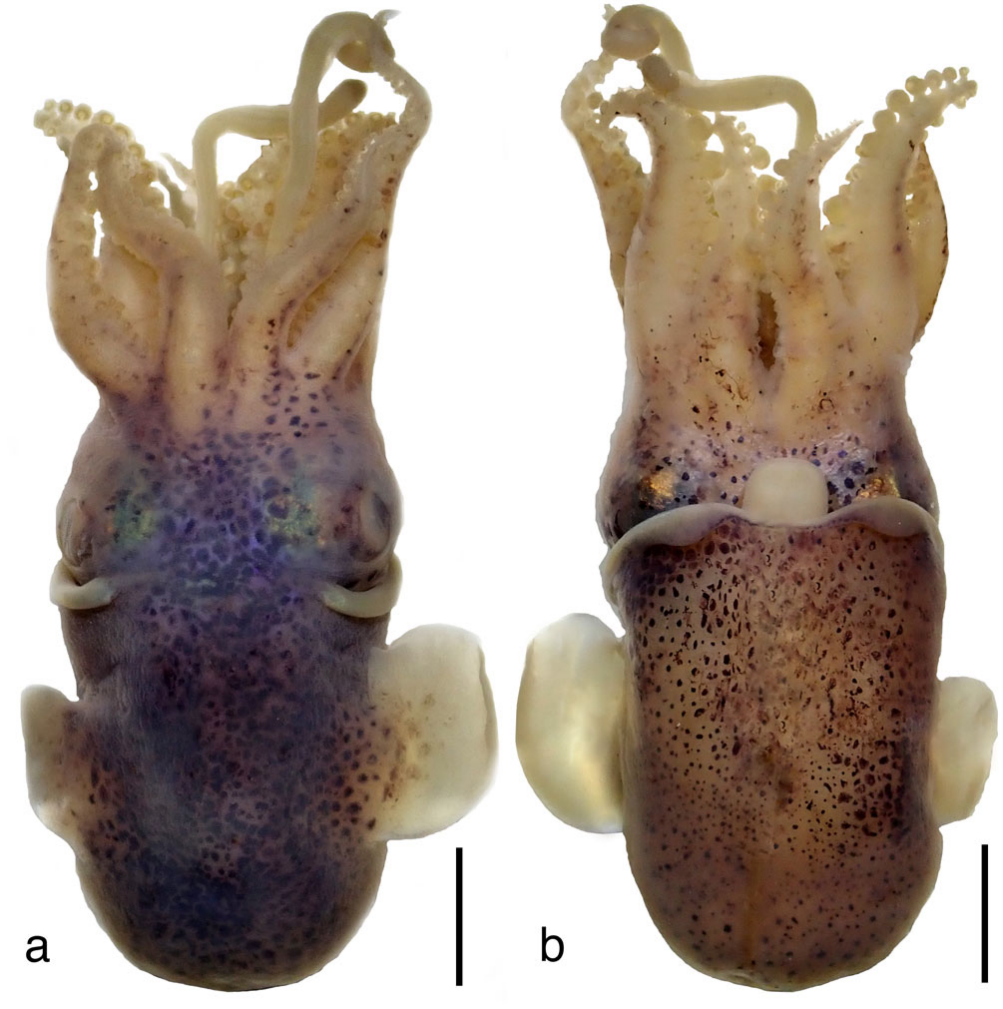
*Euprymna brenneri* sp. nov. Holotype male, 14.9 mm ML (NSMT Mo 85885): **a** Dorsal view, scale bar 5 mm and **b** Ventral view, scale bar 5 mm.

Ryukuan Type 1 is clearly distinct from other nominal Indo-Pacific bobtail squid (*Euprymna berryi* Sasaki, 1929, *Euprymna morsei*, *E. scolopes*, *Euprymna tasmanica* (Pfeffer, 1884), *S. birostrata*), and represents a distinct species. Since we did not find wild adults whose COI sequence matched Type 1 eggs and could not raise hatchlings to adults (Methods), however, we could not assert Type 1 as a new species because formal identification of bobtail squid is based on adult morphology. Type 1 hatchlings have a bilobed light organ, a broad ligament between head and mantle, and two rows of arm suckers (as in *E. pardalota*, *Euprymna phenax*, and *E. parva* (formerly *S. parva*, see below)), consistent with assignment to the genus *Euprymna*. It would be interesting to determine whether the club suckers of Type 1 have the distinctive finger-like papillae as noted for *E. pardalota* Reid^39^: Fig. 4d) and *S. parva* (Takayama and Okutani^26^: Fig. 6). Although based on the COI marker Type 1 appears to be most closely related to *E. pardalota* (from Australia and East Timor), the COI distance exceeds typical intra-specific variation^37,45^ (Supplementary Note 2). Unfortunately no RNA samples were available from this species, so we could not apply the more sensitive transcriptome analysis.

Ryukyuan Type 2 forms a clade with *Sepiola parva* from mainland Japan based on transcriptome analysis, COI marker, and morphology. Type 2 squid therefore conform in all respects to the species originally described, and recognized in the published literature, as *Sepiola parva* Sasaki 1913. We confirmed this (in the absence of the type specimen) by collecting additional specimens from Sagami Bay, close to the type locality (Methods). The identification of Ryukyuan Type 2 with *S. parva* Sasaki 1913 from Sagami Bay (redescribed below as *E. parva* comb. nov.) is consistent with dispersal of the species along the Kuroshio current that connects Zamami Island and western Okinawa in the south with Sagami Bay in the north.

Phylogenetic analysis based on both transcriptomes and COI, however, shows that type 2/*S*. *parva* is nested within the *Euprymna* clade. Thus the assignment of this species to the genus *Sepiola* renders the genus *Euprymna* polyphyletic. In our transcriptome analysis *S. birostrata* appears as the sister group to the *Euprymna* species, including all three Ryukyuan types. We conclude that Type 2/*S. parva* belongs in the genus *Euprymna* rather than *Sepiola*, based on molecular as well as morphological characters. We, therefore, propose a new generic combination, *Euprymna parva* comb. nov., for this taxon (see Systematic Descriptions). Our findings also required revision of the diagnosis for *Euprymna* (see Systematic Descriptions).

Ryukyuan Type 3 is distinct from all other bobtail squid based on both molecular (COI and transcriptome) and morphological characters. We propose below that Type 3 represents a novel species of *Euprymna*, described formally below (Systematic Descriptions).

### Molecular and temporal divergence

Pairwise synonymous substitution rates (Ks) between clades are shown in Supplementary Fig. 1. The genetic distances (Ks) within each Ryukyuan type are small compared with the distances between them. The divergence among *Euprymna* species, including Type 1, Type 2 /“*S*”*. parva*, and Type 3, is considerable (5.1–18.6%), but not as great as the divergence between *S. birostrata* and *Euprymna* species (except Ryukyuan Type 1 and Type 2/“*S*”*. parva*) which is notably higher (33.8–38.4%), consistent with the assignment of these taxa to distinct genera. The divergence between Type 1, Type 2/“*S*”*. parva* and *S. birostrata* is also high (32.6–40.5%) and is comparable to the intergeneric divergence between *S. birostrata* and *Euprymna* spp. (including Type 3).

Using a local molecular clock method and calibrations from Tanner et al.^1^, we estimate that the Indo-Pacific *Euprymna*, including Ryukyuan Types 1–3, diverged from each other 15–25 Mya (Methods). If the molecular clock is accurate, then these lineages diverged prior to the partial submergence of the Miocene land-bridge that connected eastern China to the main island of Japan ~10 Mya^46^.

## Systematic descriptions

Here, we present formal descriptions of (1) the genus *Euprymna* Steenstrup, 1887; (2) *Euprymna parva* comb. nov., formerly *Sepiola parva*; and (3) the new *Euprymna* species.

***Euprymna*** **Steenstrup, 1887**

Gender feminine. Type species, by subsequent designation, *Inioteuthis morsei* Verrill, 1881. Recent. Western Pacific and eastern Indian Oceans.

### Diagnosis

(Amended from Norman and Lu^47^ and Reid^39^, and after Bello, personal communication.) Broad ligament between head and mantle; commissure greater than one-third of head width. Transverse suckers in two or more rows on normal (non-hectocotylised) arms. Stalked suckers in six or more transverse rows on tentacular clubs. Left arm 1 hectocotylised in mature males; distally with lengthened, columnar sucker pedicels, closely packed to form longitudinal “palisades”, bearing at tip embedded toothed suckers that are partially covered by fleshy cap, number of palisades proximally equal to that of regular sucker rows but reduced toward distal tip of arm; pedicels not bearing discretely demarcated rounded suckers; basal part of hectocotylised arm with normal suckers and sometimes with 1–2 finger-like papillae in ventral sucker row, sometimes bearing tiny sucker(s). Enlarged arm suckers usually present in male and sometimes present in females. Paired kidney-shaped light organs in mantle cavity, ventral, and closely adherent to ink sac. Gladius absent.

### Remarks

Given that some *Euprymna* are now known to have biserial arm suckers, members of the genus *Sepiola* seem superficially to conform to this diagnosis. However, these two taxa (i.e., *Euprymna* and *Sepiola*) clearly differ based on molecular traits and in a number of other important characters in detail. The modification of the hectocotylus is quite distinct. In *Sepiola*, the hectocotylised left dorsal arm is thicker than the right and strongly recurved aborally in preserved specimens. The palisade columnar suckers in the hectocotylus distal portion are unique for *Euprymna. Sepiola* as well as all the other Sepiolinae genera bear regular suckers in the distal part of the hectocotylus. Some of them may be enlarged in some species, their stalks may be also lengthened and/or swollen, but no other Sepiolinae species have columnar stalks with embedded suckers at their tips. Although the suckers are positioned on enlarged and elongate pedicels in both taxa, the suckers are ovoid and discrete in *Sepiola*, while in *Euprymna* the suckers are partially capped or encased by the pedicels and the chitinous rims are usually narrow. Most remarkably, in *Sepiola*, instead of a finger-like papilla at the base of the hectocotylus (as seen in most *Euprymna* species) there is a distinct fleshy mound that may bear hook-like projections. The third arms of *Sepiola* males are thick and strongly curved orally in some species; in *Euprymna* the third arm pair of males is not swollen and recurved. *Sepiola* generally have two rows of suckers on each arm (as do some species of *Euprymna*), but some species have 4–8 rows of suckers on the distal tips of the fourth (ventralmost) arm pair. *Euprymna* may have two or more rows of arm suckers but if four or more rows are present, they are not confined to a single arm tip. In addition, the tentacular club is recurved and relatively short in *Euprymna* and longer and much less curved in *Sepiola* with a much narrower keel. The fins in *Sepiola* are large and round, while in *Euprymna* the fins are narrower and more elongate in outline.

***Euprymna parva*** **(Sasaki, 1913), comb. nov.**

(Figure 1c left, Table 1, Supplementary Figs. 1, 2c, d, Supplementary Table 2).

**Fig. 5:**
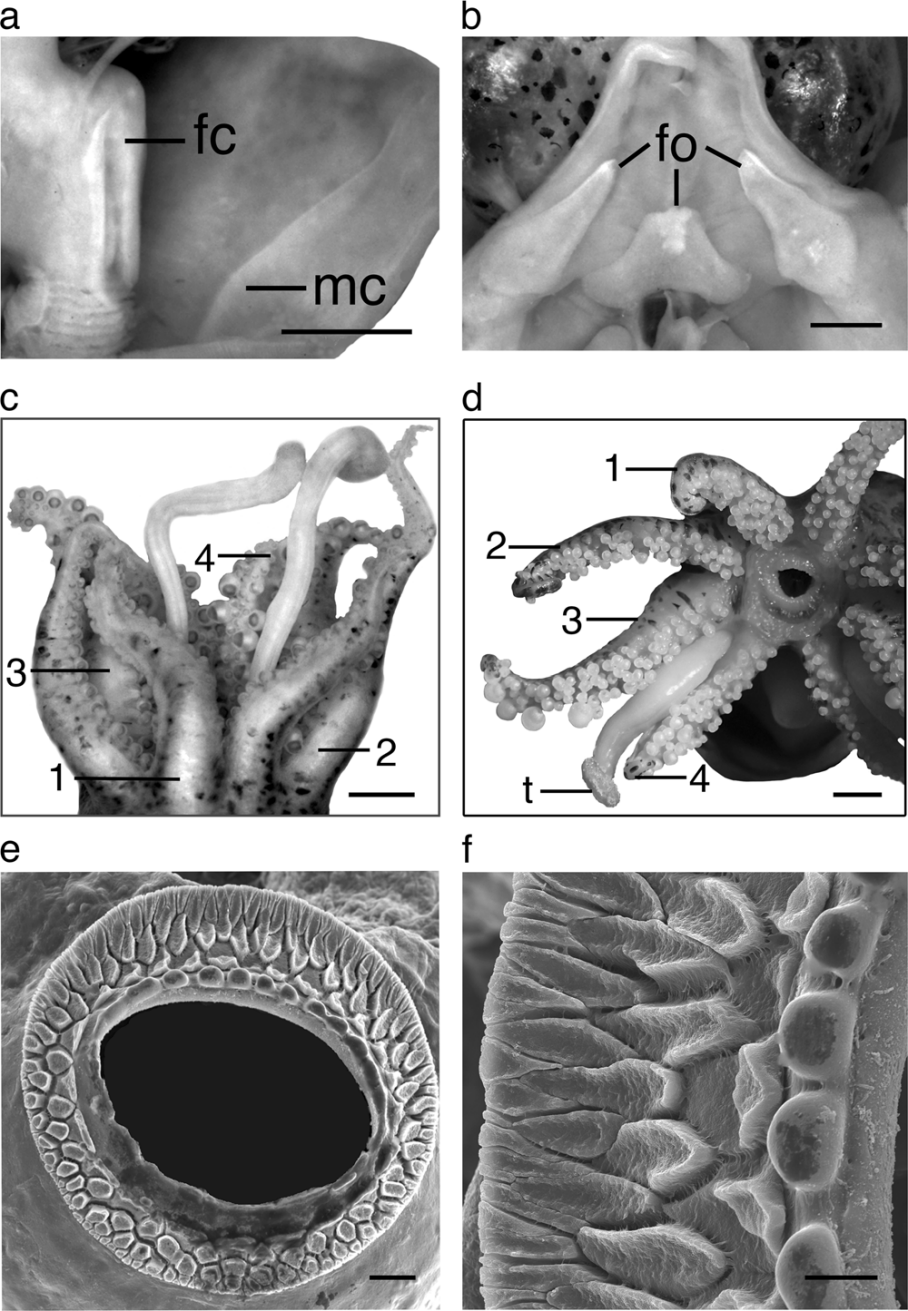
*Euprymna brenneri* sp. nov. **a** Ventral mantle dissected to show funnel-locking cartilage, fc, and mantle-locking cartilage, mc, paratype male, 22 mm ML (NSMT Mo 85891), scale bar 3 mm. **b** Funnel dissected from ventral side to show funnel organ (fo), same specimen, scale bar 2 mm. **c** Male arm crown, dorsal view, holotype 14.9 mm ML (NSMT Mo 85885), scale bar 5 mm. **d** Female right side (of animal) arm crown, oral view, paratype, 15.3 mm ML (NSMT Mo 85893), scale bar 2 mm. **c**, **d** Numbers 1–4 indicate Arms 1–4. **e** SEM Arm 4 sucker rim, paratype female, 19.5 mm ML (NSMT Mo 85889), scale bar 20 µm. **f** SEM enlargement of sucker rim shown in (**d**), scale bar 10 µm.

**Fig. 6:**
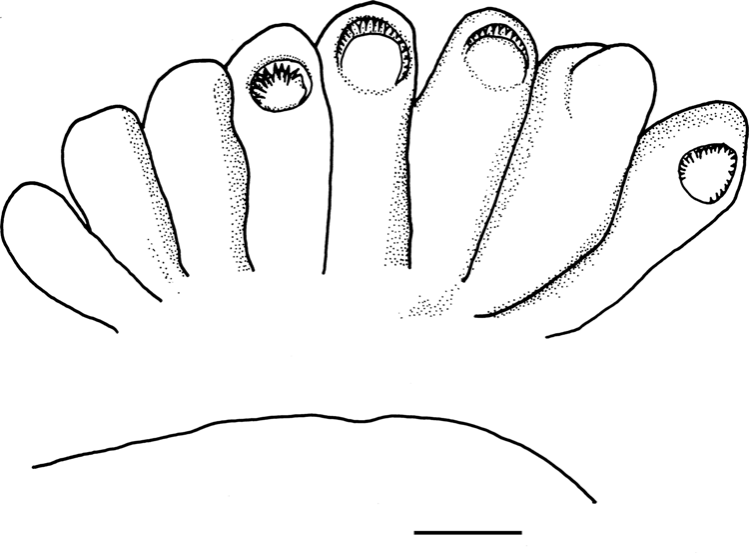
*Euprymna brenneri* sp. nov. aboral view of portion of modified part of hectocotylus (outermost sucker row) to illustrate palisade arrangement, toothed rim of palisade suckers visible at tips of (from left), suckers 4–6 and 8; cap partly obscuring chitinous sucker rim at tips of 5, 6; caps removed from (from left) sucker 4 and 8, 22 mm ML male (NSMT Mo 85891), scale bar 1 cm.

*Sepiola parva* Sasaki, 1913: 252, Fig. 4. — Sasaki, 1929: 136–137 Pl. XV, Figs. 4 and 5, text Fig. 80; Takayama and Okutani, 1992: 203–214, fig, 2, Figs. 4–6. — Okutani, 1995: 45, fig. 43. Reid and Norman, 1998: 717. — Reid and Jereb, 2005: 165–166, fig. 239.

*Inioteuthis parva* Sasaki, 1914: 595, pl. 11 Figs. 9 and 10.

Type locality: Japan, Tokyo Bay.

### Material examined

3♂ (8.5–10.7 mm ML), 3♀ (7.0–8.0 mm ML), East China Sea, Okinawa, Diamond Beach in Seragaki, 26.51N, 127.88E, <2 m, 15 June 2016, coll. J. Jolly, G. Sanchez, A. Masunaga & K. Asada (AM C.574777, Hap 3, and GenBank accession number: LC417215).

### Remarks

The correct generic placement of this taxon has been equivocal since it was first described. It was first placed in *Sepiola*, transferred to *Inioteuthis* Sasaki, 1914, then later retained in the genus *Sepiola*. Sasaki does not explain why *parva* was placed in *Sepiola* in his 1929 treatise, nor why it was referred to *Inioteuthis* in his 1914 work. Clearly, however, the features noted by Sasaki, “the nipple-like protruberance” near the base of the hectocotylus and the “peculiar and unstalked cylindrical suckers” (Sasaki^48^: 137) are characteristic of *Euprymna*. Mature males have two rows of suckers on their arms, in contrast to the four rows typically (but not always) found among *Euprymna*. This has, no doubt, resulted in its misplacement in the genus *Sepiola*, that has continued largely without question until now. However, two rows of arm suckers are also found in *E. pardalota* and *E. phenax*. The inclusion of the *E. pardalota* COI sequence in our analyses (Fig. 2) confirms its position, and that of “*Sepiola*” *parva* in the monophyletic genus *Euprymna*. Optic lobe transcriptome data (Fig. 3) clearly places *S. parva* in the monophyletic *Euprymna* clade, and the pairwise synonymous substitution rate (Ks) between clades (Supplementary Fig. 1) highlights the disjunction between *Euprymna* and *Sepiola*, providing strong support for their distinct generic status. The genetic distance data fully supports the placement of *S. parva* in the genus *Euprymna* and this evidence now permits a more robust definition of the genus based on morphological characters.

The hectocotylus (dorsal left arm) of *Euprymna* is unique among the Sepiolinae. In all genera but *Euprymna*, the hectocotylus is clearly tripartite, with a morphologically distinct basal part, copulatory apparatus, and distal part^49,50^. In contrast, in *Euprymna*, the hectocotylized arm has a bipartite form, with a proximal portion and a distal modified part. In *Euprymna* there is no distinct copulatory apparatus, instead the pedicels of the ventral suckers in the third to fourth proximal rows are modified to form 1–2 papillae in most species, sometimes bearing a vestigial sucker, whereas in all other genera the sucker pedicels forming the copulatory apparatus are more conspicuously modified (mostly horn- or hook-like). More importantly, the distal-most portion of the *Euprymna* hectocotylus bears deeply modified sucker-stalk elements: the stalks are columnar, i.e., thickened and lengthened, and appressed to each other to form palisades, and the sucker proper is reduced to a small opening surrounded by a chitinous rim, often covered by a fleshy cap and embedded in the columnar pedicel. On the contrary, in all other genera, the hectocotylus distal suckers are normal (in some cases some of them may be enlarged and/or their stalks slightly lengthened) (Bello, submitted). The very simple copulatory apparatus of *Euprymna* is considered a plesiomorphic character state^51^ in the Sepiolinae, placing this genus in a basal position within the subfamily^36,52^.

***Euprymna brenneri*** sp. nov.

(LSID: urn:lsid:zoobank.org:pub:B2D2A34E-FB8C-4D45-824E-9530986C6D44; Figs. 1c right, 4–9; Table 1; Supplementary Fig. 1; Supplementary Tables 2 and 3, and Supplementary Data)

**Fig. 7:**
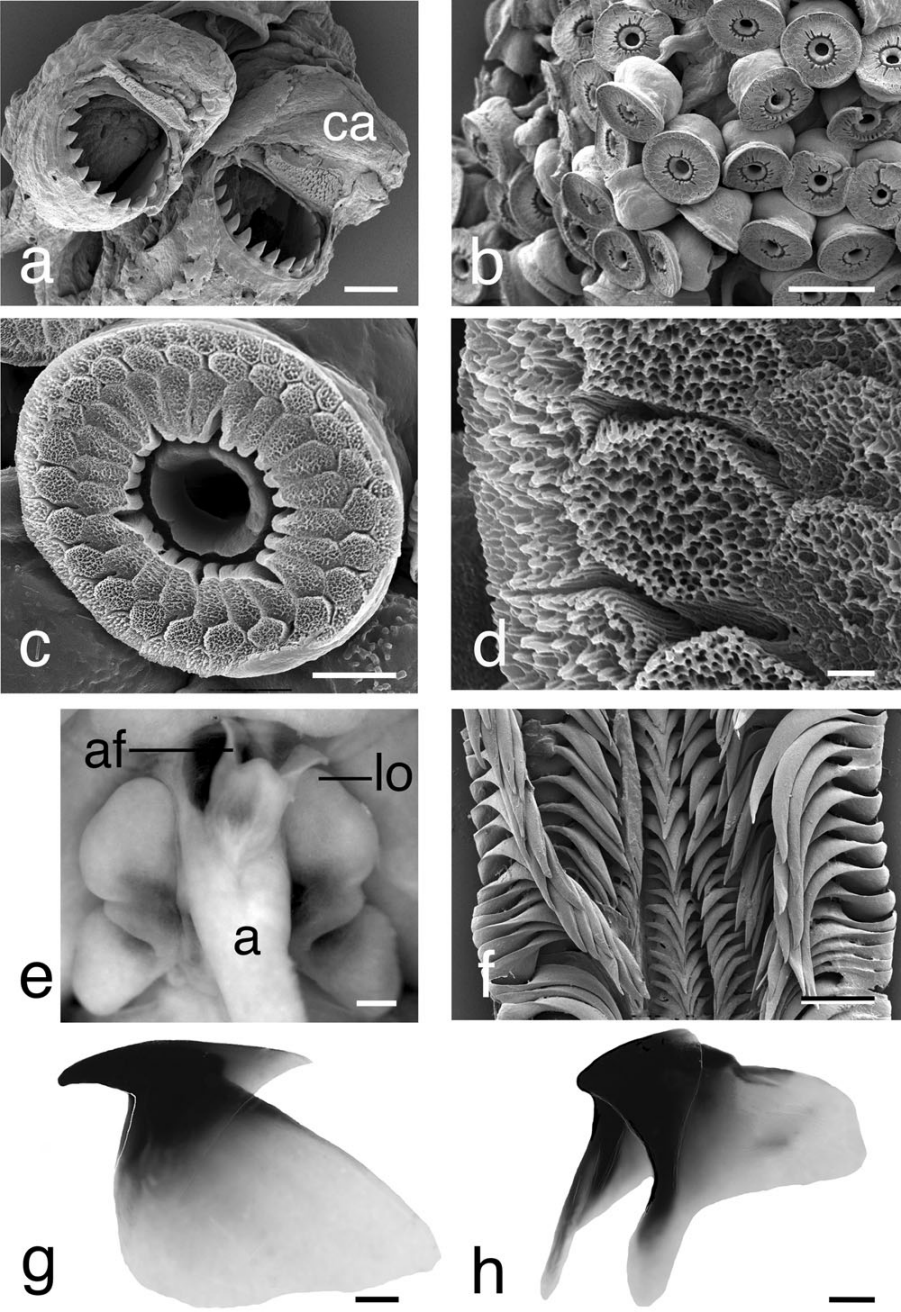
*Euprymna brenneri* sp. nov. paratype male 22.0 mm ML (NSMT Mo 85891): **a** SEM, hectocotylised right Arm 1, distal tips of two columnar suckers, showing proximal toothed sucker margins and capped portion, ca, scale bar 100 µm. **b** SEM, portion of tentacular club showing crowded minute suckers, scale bar 50 µm. **c** SEM, enlargement of tentacular sucker, scale bar 10 µm. **d** Enlargement of tentacular sucker pegs showing strongly pitted surface, scale bar 1 µm. **e** Ventral view of mantle cavity, following longitudinal incision of mantle and funnel to show anus, a; anal flaps, af, and light organ, lo, scale bar 1 mm. **f** Radula, scale bar 100 µm. **g** Upper beak, scale bar 0.5 mm. **h** Lower beak, scale bar 0.5 mm.

**Fig. 8:**
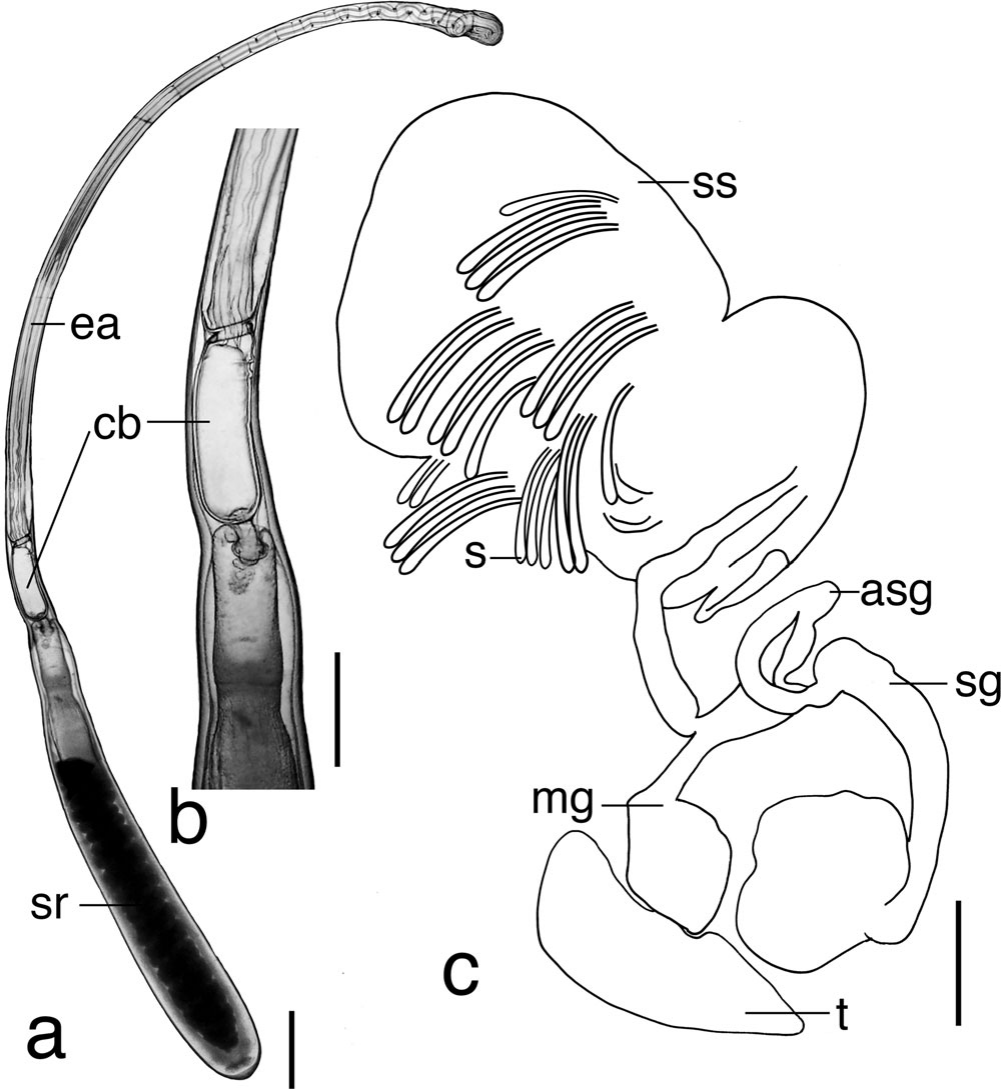
*Euprymna brenneri* sp. nov. paratype male 22.0 mm ML (NSMT Mo 85891): **a** Spermatophore, scale bar 0.5 mm. **b** Enlargement of spermatophore cement body, scale bar 0.3 mm. **c** Reproductive tract, asg, accessory gland; mg, mucilaginous gland; sg, spermatophoric gland; ss, spermatophore storage sac (thin-walled sac partially broken); s, spermatophore; t, testis; scale bar 5 mm.

**Fig. 9:**
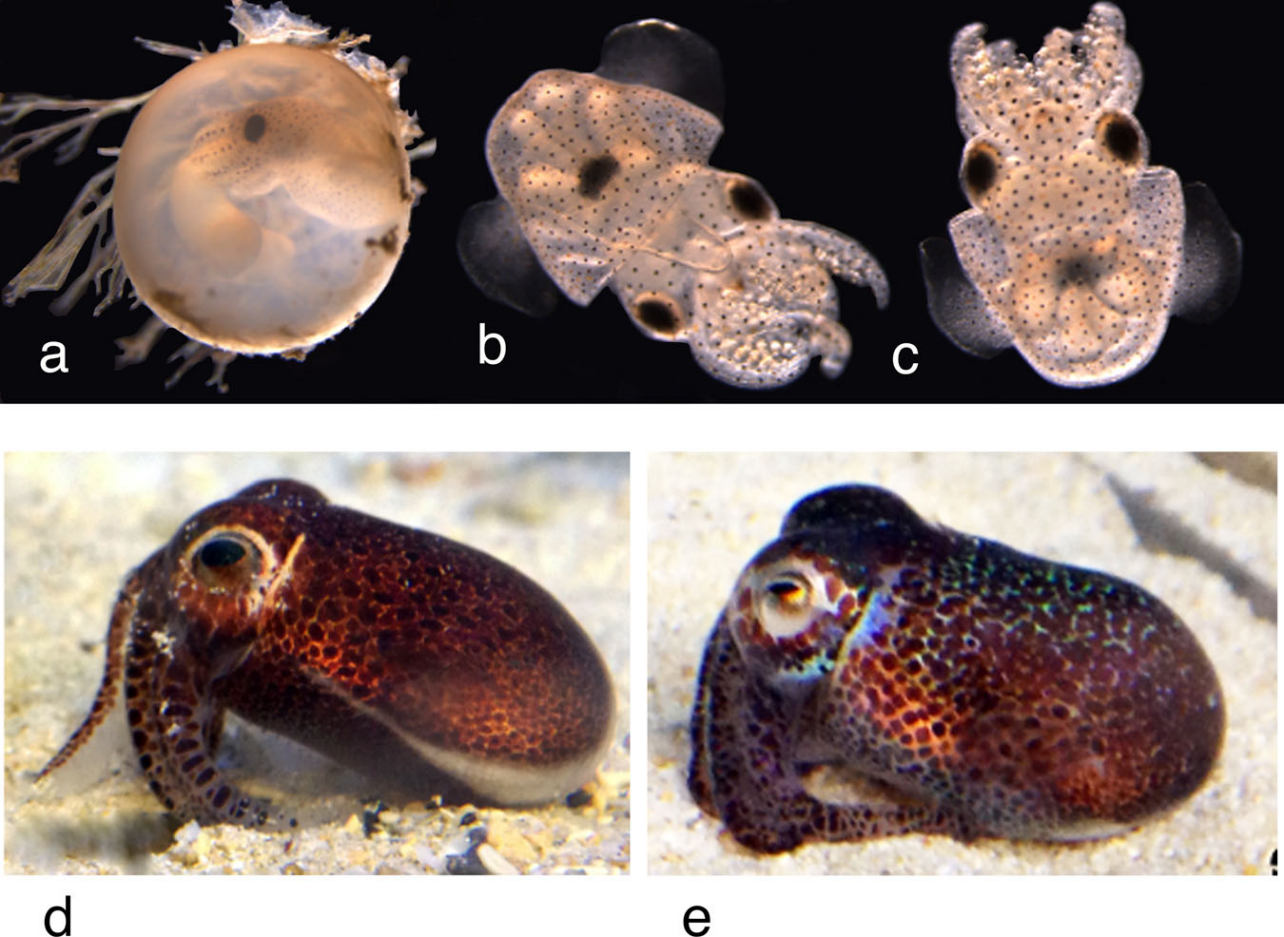
*Euprymna brenneri* sp. nov. developmental stages. **a** Developing embryo inside egg attached to yolk sac (note attachment threads on egg). **b** Laboratory-reared hatchling, ventral view. **c** hatchling, dorsal view. **d**, **e** Captive reared adults. Note arm banding clearly visible in (a and d) and concentration of blue reflective iridophores in (**e**).

### Material examined

HOLOTYPE 1♂, 14.9 mm ML, immature, Okinawa, Diamond Beach in Seragaki, 26.51N, 127.88E, 26 Apr. 2016, coll. J. Jolly & O. Simakov (NSMT Mo 85885). PARATYPES Okinawa, Seragaki, Diamond Beach, 26.51N, 127.88E, 26 Apr. 2016, coll. J. Jolly & O. Simakov: 1♂, 18.8 mm ML, (NSMT Mo 85886); 1♂, 9.0 mm ML, immature (NSMT Mo 85887); 1♂, 10.6 mm ML, immature (NSMT Mo 85888); 1♀, 19.5 mm ML, immature (NSMT Mo 85889); 1♀ 11.6 mm ML, immature (NSMT Mo 85890). Okinawa, Miyagi Is, 26.38N, 127.99E, <2 m: 1♂, 22.0 mm ML, mature, 18 Mar. 2016, coll. B. Grasse, J. Jolly, O. Simakov, S. Nyholm, K. Keunig, A. Masunaga, F. Marletaz (NSMT Mo 85891, Hap 23, and GenBank accession number: LC417234); 1♀, 9.1 mm ML, immature, 24 Feb. 2018, coll. G. Sanchez, J. Jolly, C. Sugimoto, K. Asada (NSMT Mo 85892, Hap 12, and GenBank accession number: LC417223). Okinawa, Kume Is, 23.33N, 126.81E, 1♀, 15.3 mm ML, immature, 12 Nov. 2018, coll. R. Minemizu (NSMT Mo 85893).

Notes: Only one male was reproductively mature, but due to damage to the arms and missing suckers this specimen was not selected as the holotype. No females were reproductively mature. All specimens with the exception of NMST Mo 85891 and NMST Mo 85892 were laboratory-reared from collected eggs.

### Other material examined

Syntypes: *Euprymna bursa*, Hong Kong, ♀ (34.4 mm ML), ZMH 1393, ZMH 1384 ♀ (~25 mm ML).

### Diagnosis

Small species, largest examined specimen (male, mature) 22.0 mm mantle length. Arm suckers biserial proximally and at distal tips, rest tetraserial. Males with enlarged suckers in dorsal and ventral rows of arms 3 and 4 (from ~5th to 8th sucker row from base on arms 3 and from ~7th to 9th sucker row from the base of arms 4). Hectocotylus without finger-like papillae basally. Females with enlarged suckers in ventral row of arms 3 (from ~8th to 13th sucker row from the arm base) and dorsal and ventral row of arms 4 (from ~10th sucker row counting from the basal-most row of suckers). Male enlarged suckers relatively larger than female arm suckers; suckers on ventral side of arm 3 larger than remaining enlarged suckers in both sexes. Enlarged suckers not in continuous rows, but interspersed with small suckers.

### Description

Counts and indices for individual specimens are given in Supplementary Table 3.

Species relatively small: ML males examined 8.6–22.0 mm ML, females 8.5–19.5 mm ML. Mantle short, broad, dome-shaped posteriorly (Fig. 4a, b); MWI males 63.6–98.8, females 62.1–94.1. Dorsal mantle joined to head by broad commissure, ventral mantle margin wide “m” shape (Fig. 4b). Fins oval; length approximately 50% ML; FIIa males 26.4–31.9, females 25.6–32.9; fin width about 30% ML, FWI males 23.9–34.9, females 21.6–45.8, attached dorso-laterally midway along mantle. Fins do not project anteriorly as far as anterior mantle margin (Fig. 4a, b).

Funnel conical, projects anteriorly beyond ventral mantle margin (Fig. 4b). FuLI males 52.3–75.5, females 52.3–67.2; free for most of its length, FFuI males 27.3–47.2, females 33.3–41.2. Funnel-locking cartilage long, narrow, parallel-sided with shallow groove of uniform depth and thickened rim. Mantle cartilage a long straight ridge (Fig. 5a). Funnel valve a tiny flap. Funnel organ (Fig. 5b) dorsal element broad, inverted V-shape, with concentration of glandular tissue mid-anteriorly; ventral elements with acute anterior tips.

Head broader than long, HLI males 47.0–69.8, females 51.7–65.4; HWI males 66.0–96.5, females 62.6–91.8. Eyes large, EDI males 11.6–23.6, females 14.4–20.7; ventral eyelids free. Distinct, large photosensitive vesicle on latero-posterior surface of head, posterior and ventral to eyes.

Arms, broad basally, tapered distally; order 3 > 1 > 2 = 4 or 3 > 2 > 1 = 4 (Supplementary Table 3). Arm length index of longest arm in males (ALI3) 97.3, female (ALI3) 92.3; arm keels absent or indistinct. All arms connected by relatively shallow webs, protective membranes absent. Arm sucker pedicels without lappets. Arm suckers tetraserial, with some biserial suckers at base and at distal tips of arms; spherical on normal arms (Fig. 5c, d) (hectocotylus differs). Chitinous sucker rims: infundibulum with 3–4 rows of pavement-like processes (Fig. 5e, f), peripheral sucker rim processes wedge-shaped, rest irregular with slightly raised outer margins (Fig. 5f). Chitinous inner rim of normal arm suckers without teeth, slightly crenulated on one side (Fig. 5f). Suckers on modified region of hectocotylus with toothed margins (Figs. 6 and 5a).

Males (Fig. 5c) and females (Fig. 5d) with enlarged suckers on outer row(s) of arms 3 and 4. Enlarged suckers clearly discernible in both sexes, even those of the smallest sizes (male 8.6 mm ML, female 8.5 mm ML). Male enlarged suckers larger than female arm suckers (Supplementary Table 3). Sucker counts range from 60 to 112 on each arm; Arms 2 with a greater number of suckers than other arms in both sexes.

*Males* with arm suckers in the following variable arrangement from proximal to distal end of arm:

*Right Arms 1 and Arms 2*: 5 rows biserial suckers, rest tetraserial. None enlarged.

*Arms 3*: 5 rows biserial suckers, rest tetraserial, distal arm tip with ~6 rows biserial suckers. Dorsal rows distal to sucker rows ~5–7 with 4–9 enlarged suckers interspersed at intervals with regular-sized suckers. (Larger specimens with greater number of enlarged arm suckers.) Ventral rows with ~5 enlarged suckers interspersed at intervals with regular-sized suckers.

*Arms 4*: (Fig. 5c) 5 rows biserial suckers, rest tetraserial, distal tip of arm with ~3 rows biserial suckers. Dorsal rows distal to sucker rows ~7–9 with ~3–4 enlarged suckers interspersed at intervals with regular-sized suckers. Ventral rows after sucker rows ~7–9 with ~2–7 enlarged suckers, some alternating at intervals with regular-sized suckers.

*Females* with enlarged arm suckers in the following variable arrangement from proximal to distal end of arm:

*Arms* 1: 4–5 rows biserial suckers, rest tetraserial, distal 4 rows biserial. None enlarged.

*Arms 2*: 2–4 rows biserial suckers, rest tetraserial, distal 6 rows biserial. None enlarged.

*Arms 3*: 5 rows biserial suckers, rest tetraserial, distal tip of arm with ~8–10 rows biserial suckers. Dorsal rows without enlarged suckers. Ventral rows with ~8–13 rows of normal suckers proximally followed by ~4–5 enlarged suckers alternating at intervals (either large and small suckers alternate, or two large suckers alternate with 1–2 regular-sized suckers) toward distal half of arms.

*Arms 4*: ~8–9 rows biserial suckers, rest tetraserial, distal tip of arm with 4–5 rows biserial suckers. Dorsal rows with enlarged suckers in rows ~11–15. Ventral rows with enlarged suckers in rows ~10–14.

In both sexes the enlarged suckers on arms 3 are larger than those on arms 4 and those in the ventrolateral rows are larger than those on the dorsolateral rows. The enlarged suckers on arms 3 displace the regular-sized suckers laterally. (The arrangement of enlarged suckers varies considerably among the specimens examined and it would be useful when more material becomes available to map the arrangement of enlarged suckers in mature specimens of both sexes and over a range of specimen sizes to determine whether a clear pattern of regular and enlarged suckers can be discerned.).

Left dorsal arm of males hectocotylised: from base to distal end of arm, one single sucker, ~seven transverse rows of normal tetraserial suckers, remaining suckers with swollen pedicels, that form palisade arrangement (Fig. 6), biserial with integument partially covering chitinous sucker rim in cap-like arrangement. No finger-like papillae at base of hectocotylised arm. Right dorsal arm of males with transverse rows of “normal” tetraserial suckers, with swollen pedicels.

Tentacles slender, stalks naked, semicircular in section. Club relatively short; ClLI males 18.6–33.0, females 17.9–32.9, recurved in preserved specimens, tapers to blunt end distally; sucker-bearing face convex. Suckers ~0.04–~0.08 mm diameter in center of club; arranged in ~16–24 crowded oblique rows (Fig. 7b). Swimming keel on aboral side of carpus broad, extends posteriorly well beyond carpus. Club sucker dentition (Fig. 7c, d): inner ring without teeth; infundibulum with three rows of pavement-like processes; inner rows sub-rectangular, narrowing toward central opening; middle and outer rows ovoid; irregular, with strongly crenulated and pitted surface.

Well-developed light organ present overlying and associated with ink sac (Fig. 7e). Individual lobes rectangular bulb-like anteriorly, slightly enlarged; rounded posteriorly.

Gills with 24–27 lamellae per demibranch. Buccal membrane with six lappets; suckers absent.

Radula with seven transverse rows of teeth (Fig. 7f). Rhachidian teeth simple, without cusps, triangular, slightly concave laterally and ventrally. First lateral teeth similar in size and shape to rhachidian teeth with pointed cusps displaced laterally and directed toward midline of radula. Second and third laterals with elongate bases, longer, curved. Third laterals with scythe-like teeth, longer than second laterals.

Upper beak (Fig. 7g) with pointed rostrum, hood curved, high above crest posteriorly; jaw angle approximately 90°; lateral wall edge with slight indentation. Lower beak (Fig. 7h) with blunt protruding rostrum, rostral edge obtuse, curved, without distinct inner angle; hood notch absent, wings almost straight. Distinct dark pigmentation restricted to rostrum and hood of upper and lower beaks.

Gladius absent.

Spermatophores (fully developed only in NSMT Mo 85891) approximately ½ mantle length. Sperm reservoir contains coiled sperm cord (Fig. 8a). Cement body unipartite; cylindrical, approximately uniform width, connects to sperm reservoir via a broad duct (Fig. 8a, b). Oral end of ejaculatory apparatus with 3–4 simple coils. Male reproductive tract similar in structure to congeners (Fig. 8c). Spermatophoric gland with very large, bulbous terminal portion.

Female reproductive tract: Ovary occupies large proportion of posterior end of mantle cavity and opens via single thick-walled oviduct at anterior end on left side. Nidamental glands paired, broad, located ventral to ovary toward anterior end. Inverted brownish-colored U-shaped accessory nidamental glands located toward distal end of nidamental glands. Large sac-like bursa copulatrix on animals’ left side. Spawned eggs 4 mm diameter with jelly coat and sand, 3 mm without jelly coat. Eggs are laid in clusters of more than 100, rather than individually (Fig. 1b, Type 3). Hatchlings 2 mm ML (Fig. 9a–c, Table 1).

### Color

Alcohol preserved specimens cream to maroon with large deep purple spots on dorsal and ventral head and mantle; spots larger and animal darker on dorsal surface (Fig. 4a, b); few scattered chromatophores on arms. Shiny bluish iridophores on head around eyes. Fins with large spots dorsally, close to junction with mantle, otherwise chromatophores absent from fins dorsally and ventrally. Club without pigment spots. Live adults rust brown with evenly scattered, relatively small pigment spots, darkest dorsally (Fig. 1c Type 3; Fig. 9d, e). Bright bluish iridophores around eyes, along anterio-dorsal rim of mantle, and underlie pigment spots on dorsal mantle (Fig. 9e). Arms banded with regular large spots and bars along their length (Fig. 9d) that can be seen even in embryos inside the eggs (Fig. 9a). Hatchlings translucent with evenly scattered chromatophores. Juveniles dark brown.

### Habitat

Adults were found in sandy near-shore shallow waters, less than 2 m in depth, among corals and rocks. Eggs were found in rocky areas near coral reefs in depths of 8–18 meters.

### Type locality

Japan, Okinawa, Seragaki, Diamond Beach, 26.51N, 127.88E.

### Distribution

Japan: Okinawa Prefecture Seragaki, Diamond Beach 26.51N, 127.88E; Oura Bay, 26.53N, 127.74E; Miyagi Island, 26.38N, 127.99E; Ishigaki Island, Oganzaki 24.44N, 124.07E; and Kume Island, Northern Hatenohama Beach (26.35°N, 126.86°E) (Fig. 1). Taiwan*: off Penghu waters on the west-northern side of Sha-kang Fishing Harbor 23.60N, 119.62E. Depth range 2–18 m. (*Based on COI obtained from a single immature specimen.)

### Etymology

The species is named in honor of the pioneering geneticist and Nobel Laureate Dr Sydney Brenner, founding president of the Okinawa Institute of Science and Technology. We also propose the common name Brenner’s bobtail in English and Burenā-mimika in Japanese.

### Remarks

The largest male (22 mm ML) (and the only fully mature specimen) examined had damaged arm tips and most suckers missing. However, the arrangement of enlarged suckers was clear from the sub-mature specimens.

Prior to this study fifteen species of *Euprymna* were recognized^25^, although three of these (*E. bursa* Pfeffer, 1884, *E. pusilla* Pfeffer, 1884 and *E. schneehagenii* Pfeffer, 1884) are considered doubtful species by Norman and Lu^47^. Among the nominal species of *Euprymna*, the species whose geographic range most closely encompasses the known range of *E. brenneri* includes *E. berryi*, which has been found in warm temperate coastal waters from China and Taiwan, south to Hong Kong and Japan^53^, and *E. morsei*, which is sympatric with *E. berryi* over its range and also occurs as far south as the Philippines and Indonesia. Both, however, differ in morphology and in molecular traits (Figs. 2 and 3) from *Euprymna brenneri*. These three taxa (*E. berryi*, *E*. *morsei* and *E*. *brenneri*) differ in their COI and transcriptome signatures. Both *E. berryi* and *E. morsei* have enlarged suckers in males on the second arm pair (on the ventral margin in *E. morsei* and the dorsal and ventral margins in *E. berryi*), while *E. brenneri* does not. Kubodera and Okutani^32^ described *E. megaspadicea*, found in deep waters (200 m) of Nago Bay off Okinawa. While no sequence data currently exists for *E. megaspadicea*, it is clearly morphologically distinct from *E. brenneri*. In *E. megaspadicea* the hectocotylised arm is longer than the opposing arm, and the hectocotylus contains a sharp lateral inward groove not seen in other *Euprymna* species.

As part of this study we examined the type specimens of *E. bursa* (ZMH RK 1384 and RK 1393, both females, approximately 25 mm ML and 34 mm ML respectively) from Hong Kong. *Eurymna bursa* differs from *E. brenneri* in that none of the arm suckers are enlarged (luckily these remain attached to the arms of the *E. bursa* types, enabling this comparison to be made). In addition, *E. bursa* has a greater number of arm suckers (102–128) and the median component of the funnel organ of *E. bursa* is spade-shaped, straight posteriorly, and not indented. (Whether *E. bursa* is a valid species awaits the examination of males from the type locality; here we verified that *E. brenneri* was not referable to this species—particularly important given their geographical proximity.)

Of the remaining *Euprymna* species (which now also includes *E. parva*), the results of the COI analyses (Fig. 2) indicate that *E. brenneri* (i.e., Ryukyu Type 3) belongs in a clade distinct from *E. berryi*; *E. hyllebergi*; *E. morsei*; *E. pardalota*; *E. scolopes*, *E. tasmanica, E. albatrossae* Voss 1963, and *Euprymna* sp. Type 1. Transcriptomes separate *E. brenneri* from *E. berryi*, *E. morsei*, *E. parva*, *E. scolopes*, and *E. tasmanica* and *Euprymna* sp. Type 1 (Fig. 3). These differences are also supported by morphological traits: no other *Euprymna* taxa are yet known to include females with enlarged suckers. In addition, male *E. albatrossae*; *E. berryi*, *E. megaspadicea, E. morsei, E. scolopes*, *E. stenodactyla* Grant, 1833, and *E. tasmanica* have enlarged suckers on the second arm pair of males, which is not the case for *E. brenneri*.

*Euprymna brenneri* does, however, have enlarged suckers on arms 3 and 4. Males and females have enlarged suckers on the ventral row of the third arm and the dorsal and ventral rows of the fourth arms, with no enlarged suckers on the first and second arms. This is the first time a female *Euprymna* has been identified with large suckers. Female members of this genus are notoriously difficult to identify based on morphology, so the discovery of this character is a valuable one. Of the other nominal species, the enlarged sucker arrangement in male *E. brenneri* is most similar to that of *E. hoylei* Adam, 1986, but this species (in addition to all other nominal *Euprymna* with the exception of *E. brenneri*) have 1–3 enlarged finger-like papillae on the proximal end of the hectocotylised arm. *Euprymna hoylei*, described from the Sulu Archipelago, like *E. brenneri*, has no enlarged suckers on the second arm, however, *E. brenneri* males possesses approximately eight very large suckers on the third arm compared to a smaller number described for *E. hoylei* (3–4). Female *E. hoylei* do not have enlarged suckers. In addition to the presence or absence of enlarged suckers on particular arms, the enlarged suckers in both sexes of *E. brenneri* are not located close to the base of the arms, as seems to be the case in other *Euprymna* species, but at some distance distal to the arm bases.

## Discussion

Here we report three distinct species of bobtail squid living in the waters of the Ryukyu islands, based on differences in egg mass, morphology, mitochondrial COI and transcriptome-based molecular markers. Moreover, we identify a novel bobtail squid species, *E. brenneri*; document that the species known as *Sepiola parva* should in fact be classified within the genus *Euprymna*, Steenstrup 1887, and close its life cycle in laboratory culture; provide an updated definition of the genus *Euprymna* and *E. parva* comb. nov.; update the DNA barcoding data set of bobtail squid; and provide a set of useful genes from the optic lobe of bobtail squid for future comparative and phylogenetic studies.

### Broader perspectives on Sepiolid phylogeny

Our analysis of diverse bobtail squid supports the monophyly of the genus *Euprymna* and the genus has been redefined based on morphological characters (Systematic Description). We find that four rows of arm suckers is, however, not a defining character for the clade, and the group includes at least four members with two rows of arm suckers: *Euprymna* sp. Type 1, *E. pardalota, E. parva*, and *E. phenax*. This undoubtedly resulted in the original misplacement of *E. parva* in the genus *Sepiola*. Evidently the two-row state is ancestral for *Euprymna*, and is retained in *E. parva* and *Euprymna* sp. Type 1 (observed in hatchlings). The four rows found in most of the remaining *Euprymna* (*E. stenodactyla* has 6–8), including *E. brenneri* and other *Euprymna* spp. in Fig. 3, is a derived state.

The genus *Sepiola* originally described by Leach^54^ united Atlantic and Mediterranean species of bobtail squid. It was later extended by Sasaki^55^ to include the Indo-Pacific species, *Sepiola parva*, *S. birostrata* and later, *S. trirostrata* Voss, 1962. In our analysis (and in Lindgren et al.^42^, Allcock et al.^56^, and Lindgren et al.^57^) with fewer species but more molecular characters, this extended version of the genus *Sepiola sensu* Sasaki appears to be polyphyletic. Also, in our COI tree (Fig. 2) *Sepiola birostrata* from Japan is only distantly related to the *Sepiola* species from the Mediterranean, and the North and Baltic Seas. This latter group is, in turn, interspersed among species of the genus *Sepietta* and *Heteroteuthis*. A critical reexamination of these relationships is needed.

The complex modification of the basal part of the hectocotylus characteristic of *Sepiola* (and indeed other members of the subfamily), albeit in different degrees, appears to be an ancestral character of a broader clade that includes the Atlantic-Mediterranean *Sepiola*. The genus *Euprymna* evidently arose from within this broader clade, accompanied by a simplification of the copulatory apparatus. Takayama and Okutani^26^ have also noted that the hectocotylised arm suckers have toothed margins in *E. parva*, but have smooth margins in *Sepiola birostrata* with which it is compared. Interestingly, as mentioned above, they also note that the inner two rows of the infundibulum of the club sucker rims bear rows of comb-like papillae in *E. parva* (referred to as *S. parva* in Takayama and Okutani^26^: Fig. 5) that are identical in appearance to those seen in *E. pardalota* (Reid^39^: Fig. 4d). It would be worthwhile to examine this character among other *Euprymna* species in a more comprehensive review of the genus.

Clear species misidentifications in the literature are also evident from our COI tree (see also Supplementary Note 1), and should be reconciled by further study. For example, samples from three *Sepiola* species (*S. robusta* Naef, 1912, *S. intermedia* Naef, 1912, and *S. affinis* Naef, 1912), all collected at Banyuls-sur-mer, have nearly identical COI markers together with a sample deposited as *Heteroteuthis dispar* (0–0.35% divergence). This limited nominally interspecific COI variation, however, is comparable to variation seen within many other species of the Sepiolidae (e.g., our Ryukyuan bobtail species), and suggests the need for a critical reexamination of these Mediterranean taxa to determine whether this seeming anomaly is due to misidentification, conspecificity among some taxa, recent divergence or a low mutation rate of this loci in some of these taxa. Conversely, we find extensive intra-specific COI variation within *E. tasmanica* from Australia, *E. albatrossae* from the Philippines and within the tropical bottletail squid *S. kochii* from Taiwan, Okinawa, and Japan.

These observations demonstrate either that molecular variation within bobtail squid species can be unusually large, or that some species will need to be further subdivided to reflect molecular variation within purported taxa. For example, Norman and Lu^47^ tentatively gave the distribution of *E. tasmanica* (with the type locality given by Pfeffer^58^ only as Bass Strait) as extending from the Great Australian Bight (southern Australia), Tasmania and extending along eastern Australia, possibly to as far north as Moreton Bay, Queensland. They recognized that an undescribed taxon with the same configuration of enlarged arm suckers occurs in northern Australia. The COI data presented here was obtained from supposed *E. tasmanica* over a wide distributional range, including Western Australia and New South Wales, so it may comprise more than a single species, particularly as many Australian marine taxa are southern Australian endemics. This taxon needs to be critically reexamined. The same may be true for animals ascribed to *Sepidarium kochii*.

Prior to the advent of molecular tools, *Euprymna* had been a particularly difficult group for morpho-taxonomists. Morphological redescription of all nominal *Euprymna* species to include additional characters for direct comparison (in addition to molecular traits) is essential. The reliance on the arrangement of enlarged arm suckers as almost the only distinguishing character is, of course, problematic. Future studies could usefully include additional characters such as sucker rim ultrastructure, spermatophore structure, and ontogenetic changes in enlarged sucker arrangement (i.e., to determine how fixed, or otherwise, is the pattern of enlargement at different stages of maturity), all of which appear to be differ to varying degrees but hard to compare at this time among all nominal species.

The dramatic differences between intra- and interspecific COI variation across the sepiolid tree point to the need for a comprehensive reevaluation of bobtail squid with both sequence markers and morphology to characterize the diversity and biogeography of this widely dispersed group. We have seen that limited RNA sequencing from optic lobe transcriptomes easily provides large numbers of robust molecular characters and can be used similarly in other group of cephalopods. Our transcriptome resources provide a foundation for comparative genomics and biology within this diverse group of cephalopods. Finally, the identification of three species of Ryukyuan bobtail squid raises interesting questions about the possible differing ecological roles played by these species.

## Materials and methods

### Collections

We surveyed bobtail squid along the Ryukyu archipelago from shallow waters to ~20 m depth. Sepiolid squid eggs and, where possible, adults, were obtained from several sites in Okinawa Island (from both the East China Sea and Pacific Ocean, from March to November in 2016 and 2017, and February to August in 2018), from one site in Ishigaki Island (July 2016), from one site in Kume Island (November 2018), and from Zamami Island (October 2016) in the Kerama Islands to the south (Fig. 1a). More sampling was done on the East China Sea side than the Pacific coast. Less extensive sampling around the Ryukyu islands was also conducted from February to November 2017 and February to August 2018. We also collected *Sepiola parva* in Sagami Bay, Tokyo (July 2017).

Adults were collected at night by hand while wading, snorkeling, or diving on SCUBA in shallow water (<2 m) off Okinawa at Miyagi Island (26.38N, 127.99E), Diamond Beach at Seragaki (26.51N, 127.88E), Motobu (26.60N, 127.91E), Sunabe Sea Wall (26.32N, 127.74E), Zamami Island (26.22N, 127.30E), and Kume Island (23.33N, 126.81E). Collection location details are shown in Table 2. Live animals were immediately brought to the culture facility at OIST (Okinawa Institute of Science and Technology) and acclimated for one hour by gradually replacing source water with aquarium water.

Eggs were collected during the day between 6 and 18 m at Seragaki (26.51 N, 127.88E), Gorilla Chop in Sakimotobu (26.63N, 127.88E), Motobu in the north of Nago Bay (26.60N, 127.91E), Mizugama (26.36N, 127.73E), Oura Bay (26.53N, 128.07E), Oganzaki at Ishigaki Island (24.44N, 124.07E), and Sunabe Sea Wall (26.33N, 127.74E) using SCUBA. Collections were made from March to November 2016 and unhatched eggs were not found after August. Stones were flipped at depths from 2 to 30 m, and all stones with eggs attached were found between 6 and 18 m.

Eggs were gently removed to a plastic container that was kept open upon ascending. They were then immediately taken to aquaria at OIST and acclimated as described for adults above. Eggs were kept in round containers with mesh bottoms inserted into two litre aquaria and exposed to a continuous water flow.

As a further aid in the sequence-based characterization of bobtail species, we also collected new samples from several previously described species: fourteen *E. berryi* from mainland Japan and Taiwan; five *Euprymna scolopes* from Honolulu, Hawaii (provided by Spencer Nyholm); five *Sepiola parva* from the Sagami Bay in Japan; seven *Sepiola birostrata* from Osaki Shimojima, in the Seto Inland Sea of Japan, Hyogo Prefecture, and Hokkaido Prefecture in the Japan Sea; and four putative *Euprymna morsei* from Mie Prefecture, Japan (Pacific Ocean) (later confirmed by sequence analysis, see below). One additional individual of the later species was obtained from Izu Chuo Aqua Trading Co., Ltd., and kindly provided by the Miller Unit at OIST. For use as outgroup taxa, we collected four bottletail squid, *S. kochii* (Sepiadariidae), from Mie prefecture, Okinawa, and Taiwan; and obtained one pyjama squid *S. lineolata* (Sepiadariidae) as a gift from the Monterey Bay Aquarium (captive bred). The identities of these samples were confirmed based on existing available molecular makers, and morphology. A complete list of COI samples used in this study is shown in Supplementary Table 2.

### Collection of new specimens of *Sepiola parva* (*E. parva* comb nov.)

Type 2 squid conform in all respects to the species originally described, and recognized in the published literature, as *Sepiola parva* Sasaki 1913. According to Gleadall^59^, however, the type specimens of this species (reportedly housed in the MSUT (University Museum, University of Tokyo)) are missing and are presumed lost. For sequence and morphological comparison, we collected new specimens from Sagami Bay, close to the type locality of *S. parva* (Tokyo Bay), and confirmed their identity by COI and transcriptome markers (Figs. 2 and 3).

### Culturing methods

A semiclosed system of five 70 l tanks, five 2 l tanks, and 100 l sump was used. An ultraviolet sanitizing light, crushed coral, mesh filter, two protein skimmers, and chiller were used to maintain water quality. Light emitting diode (LED) aquarium lights provided a 12:12 light:dark photoperiod. A dim blue LED light was illuminated during the dark cycle to mimic moonlight. Sand-filtered seawater was provided weekly from a local source supplied by OIST Animal Support and was maintained at 24 °C. Water changes of 15% were performed weekly. Salinity averaged 35 parts per thousand (ppt) but ranged between 33–36 ppt. The pH was maintained between 8.00 and 8.15. Alkalinity, ammonia, and nitrate were measured three times per week using qualitative tests. Ammonia never exceeded 0.15 ppm and nitrate never exceeded 5 ppm. Sand was collected from where the animals were obtained and washed with deionized water prior to adding to the aquaria.

Paralarvae and juveniles were fed live wild-caught mysid shrimp, *Neomysis* sp. *ad libitum* twice daily. Mysids and wild-caught *Palaemon* sp. and *Caridina* sp. were provided to juveniles and adults.

### Laboratory culture of Ryukyu type 1 (*Euprymna* sp.)

We found Ryukyuan Type 1 eggs near both Okinawa and the Ishigaki Islands. Type 1 hatchlings transcriptomes are distinct from those of known adult Indo-Pacific bobtail squid. In laboratory culture, Type 1 hatchlings are smaller than hatchlings of the other two types of Ryukyuan bobtail squid. Although hatchlings swim immediately upon hatching, and some feeding behavior was observed, no hatchling survived past 10 days. It is possible that in our culture they hatched prematurely, or that once hatched they lacked some environmental cue to properly develop their predatory behavior.

### Laboratory culture of Ryukyu type 2 (*E. parva* comb. nov.)

We found Ryukyuan Type 2 bobtail squid in Okinawa (along the East China Sea), the Zamami Islands, and Miyagi Island (on the Pacific Ocean side). We raised Ryukyuan Type 2 to maturity in our laboratory, following culturing conditions described for other bobtail squid. Modifications include the use of a semiclosed system maintained at 23 °C, and an exclusive diet of mysid shrimp (*Neomysis* sp.). The life cycle, from hatching to sexual maturity to hatching of progeny, took approximately three months. The closure of the life cycle of Ryukyuan Type 2 will enable comparative analysis of behavior and physiology between the mainland Japanese and Ryukyuan populations. Further, *E. parva*’s small size at maturity, abundance throughout the year in easy to access areas, and simple diet promote this species as a viable option to study cephalopods in laboratory settings.

### Laboratory culture of Ryukyu type 3 (*E. brenneri*)

We found Ryukyuan Type 3 bobtail squid in shallow waters off Okinawa Island (Seragaki on the East China Sea, and Oura Bay and Miyagi island on the Pacific coast), off Ishigaki Island to the south, and off the Penghu coastal waters of Taiwan (Fig. 1a). We raised Type 3 hatchlings to maturity in the laboratory, and observed mating behavior, but no eggs were laid. In captivity, hatchlings exhibit a paralarval stage similar to *E. scolopes* hatchlings^20^ and swim at the surface of the water column in a positive phototaxic manner both during simulated day and night photoperiods. Squids maintain a dark coloration with expanded chromatophores, except while preparing to capture their prey, at which point chromatophores contract and squids appear colorless. Adult mysids longer than 8 mm were hunted successfully on the fourth day post hatching. Squid were observed to seize they prey with their tentacles from below, holding it with their arms while eating. Settling and burying behavior was first observed at day five. Hatchlings periodically entered the water column until day 25 when all squids exhibited completely benthic lifestyles. Sexual dimorphism was visibly apparent at day 65 based on visual observation of the hectocotylus compared with non-hectocotylised left arm 1 in males. From day 65 males and females were separated in our cultures and brought together to test for mating behavior. Mating was achieved on day 83 and carried on for approximately 60 min. Mating behavior resembled previously described behavior in other *Euprymna* spp.^20,60,61^. After the last female died, unfertilized eggs were found inside her ovary. The longest-lived squid was a male that died on day 99.

### COI sequencing and phylogeny

Genomic DNA was isolated using the MagAttract HMW DNA kit from small tissue samples taken from the arms of hatchlings or adults and the fins of randomly selected specimens in our tanks. The universal COI primers^62^ LCOI1940 5′-ggtcaacaaatcataaagatattgg-3′ and HCOI2198 5′-taaacttcagggtgaccaaaaaatca-3′ were used to obtain fragments of more than 574 bp. Polymerase chain reaction (PCR) was performed in a Bio-Rad T100^™^ Thermal Cycler with an initial denaturation at 94 °C for 1 min. followed by 30 cycles of 94 °C for 30 s., 42 °C for 1 min, 72 °C for 1 min; and a final extension at 72 °C for 10 min. PCR reactions were carried out in a total volume of 10 µL containing 3.0 µL of DNA template, 1.0 µL of 10× Taq Buffer, 0.8 µL of dNTPs (10 µM), 0.2 µL of each primer (10 µM), 0.25 U of Ex Taq (TAKARA) and 4.8 µL of D2W. PCR products were cleaned with ExoSAP-IT (Affymetrix/USB corporation) and then sequenced using a BigDye v3.1 Terminator Sequencing Kit (Applied Biosystems) using the LCOI1940 primer on a Genetic Analyzer (ABI 3130xl, Applied Biosystems).

In addition to the sequences we generated here, sepiolid COI sequences deposited in Genbank from several other studies were integrated into our analysis (Supplementary Table 1). A COI sequence from *E. pardalota* was included. The *E. pardalota* sequence data were obtained from a specimen collected in East Timor (note not the type locality) and lodged in the Australian Museum Collections (AM C.476100.002, EBU 80020).

Sequences were aligned in MAFFT v7.158b^63^ and the phylogenetic reconstruction was performed with IQ-tree software^64^ using maximum likelihood (ML) with 1000 ultrafast bootstrap replications^65^ and the best fit model determined by ModelFinder^66^.

### RNA isolation and sequencing

We dissected the optic lobes from randomly selected hatchlings of our three Ryukyuan bobtail squids species and *E. scolopes* egg masses in our tanks, an embryo of *S. lineolata* and juveniles of *Sepiola parva*. Mantle muscle was also dissected from adult *Sepiola birostrata*, *S. kochii*, *Euprymna berryi*, and female putative *Euprymna morsei*. Details of the samples, collection locations, and tissue used are shown in Supplementary Data.

Total RNA was extracted with the RNeasy micro kit (Qiagen) and quantified by Qubit fluorometer (Invitrogen). RNA quality integrity numbers (RIN) were obtained using the Agilent 4200 TapeStation. cDNA was synthesized and barcoded, and RNAseq libraries were produced with the Illumina NeoPrep TrueSeq stranded mRNA library prep kit. Ten barcoded strand-specific libraries were pooled and sequenced in a single lane of Illumina HiSeq 4000 (paired end 2 × 150 bp sequences). In total, four lanes were sequenced.

In addition to the sequences generated here, we also used RNAseq data from *E. tasmanica* donated by Prof. Jan Strugnell from James Cook University.

### Transcriptome analysis and orthology determination

Raw reads were visualized with FastQC (http://www.bioinformatics.babraham.ac.uk/projects/fastqc/). Removal of low quality reads and trimming of adapter sequences were performed in Trim Galore v. 0.4.0 (http://www.bioinformatics.babraham.ac.uk/projects/trim_galore/) with cutadapt^67^. *De novo* transcriptome assemblies were performed using Trinity^68^. We also used CD-HIT-EST^69^ to remove putative haplotype redundancy and keep the longest contigs. Open reading frames (ORFs) and coding sequences (CDS) were identified with Transdecoder v3.0.1 (http://transdecoder.github.io). Orthology assignment was performed with OMA stand-alone v.1.1.2^70^ using default parameters and an all-against-all pairwise amino acid sequence comparison run in parallel on the Okinawa Institute of Science and Technology HPC cluster. From the OMA output, we used (1) the “OMA groups” or orthogroups provided in fasta format, where all pairs of genes within one group are orthologous to each other and each group contains one sequence per species, and (2) the all *vs*. all pairwise orthology inference in text format, from which the list of 1:1 orthology relationships were used for the Ks (synonymous substitution) distance determination (see details below).

### Matrix construction and phylogenetic analysis

For phylogenetic analysis we extracted 452 orthogroups containing all 40 individuals from a total of 1325 unique orthogroups (containing at least our 19 Ryukyuan individuals) generated by OMA. Four different matrices were constructed using coding and peptide sequences, each pair set including 452 orthogroups (no missing taxa) and 1417 orthologous groups (including missing taxa). For this latter group, for each species we included the top 50% of individuals based on their orthogroup content. Amino acid and CDS were aligned in MAFFT v7.158b and trimmed using Gblock v0.91b^71^ allowing at most 50% gap positions prior to concatenation. The resulting data matrices consist of 136,333 (no missing data) and 413,030 (no missing data) positions for the amino acid and CDS set with no missing taxa, while on 514,512 (5.40% missing data) and 1,558,759 (5.39% missing data) positions for the amino acid and CDS set with missing taxa.

Maximum likelihood analyses were performed for the four matrices by using the IQ-tree software with an ultrafast bootstrap approximation of 1000 replicates, and the best model estimated by ModelFinder (-m MFP + MERGE) with the relaxed clustering algorithm (-rcluster 10)^72^ under the Bayesian Information criterion.

Bootstrap values above 95% were considered as highly supported. Trees based on nucleotide sequences showed almost all clades with high support (including all our species clades supported with 100% bootstrap).

Bayesian inference was performed in Exabayes v1.5^73^ using two independent runs with four coupled chains each and the GTR + Γ model for each gene. Only the data matrix based on CDS with no missing taxa were used for the practical reason mentioned above. Each independent run was continued for 1 million generations, sampling every 500 until the average standard deviation of split frequency was <1%. The first 25% of samples were discarded as burn-in every 500 generations and proper sampling post burn-in was evaluated by an effective sample size (ESS) of >500.

### Pairwise synonymous distance (Ks) calculation

Intra- and interspecific synonymous distance (Ks) were calculated using the Yang-Nielsen estimates from the yn00 program of PAML package 4.8^74^. Using the OMA all vs. all pairwise orthology inference from Ryukyuan species and their congeners, we selected the median Ks values from each of the 1:1 orthologous gene pairs.

### Divergence time estimation

Divergence times among the Ryukyuan species were estimated using the r8s v1.80 program^75^ with a local molecular clock. We used the maximum likelihood tree with branch length estimated from the IQ-tree software, based on ultrafast bootstrap with 1000 replicates. We included RNA-seq data of *Idiosepius paradoxus* Ortmann, 1888 (SRR2984343) and *Sepia esculenta* Hoyle, 1885 (SRR1386223), with a calibration of 174 Mya, the midpoint of the range (129, 219 Mya) from Supplementary Fig. 4 of Tanner et al.^1^, for the splits between Sepiidae with Idiosepiidae and the Sepiolidae.

### Morphology

Terminology, measurements, indices, and abbreviations for anatomical structures referred to in the new species description follow Roper and Voss^76^, with a few minor differences (Supplementary Table 4). In the current paper, ASC refers to the total number of suckers on each designated arm (this abbreviation refers to the number of suckers on the basal half of each arm in Roper and Voss^76^, with ASCT used for the total number of arm suckers). All measurements are in millimeters (mm). Measurements and counts for individual mature and sub-mature specimens are presented in Supplementary Table 3; the range of values for each character is expressed in the description as: minimum–maximum. The values for each sex are given separately. Only large specimens were measured, but only one (NSMT Mo 85891), a male, was fully mature.

Other abbreviations: AM, Australian Museum, Sydney; NSMT, National Museum of Science and Technology, Tokyo, Japan; ZMH, Zoologisches Museum, Universitat Hamburg.

For scanning electron microscopy, dissected tissues were mounted, then air dried and examined after gold coating in either a JEOL JSM 7100 FESEM Field Emission Scanning Electron Microscope, or a JOEL 6480LA SEM Tungsten Variable Pressure Scanning Electron Microscope.

### Reporting summary

Further information on research design is available in the Nature Research Reporting Summary linked to this article.

## Supplementary information

Supplementary Information

Description of Additional Supplementary Items

Supplementary Data

Reporting Summary

## Data Availability

Study accession number for RNA sequencing: SRP157062. Accession numbers for COI sequences: LC417211–LC417235. Alignments, best partition scheme and models are available in Figshare https://figshare.com/s/94a59f3ce82cacabaa7c.
