# Supplementary material for: New bobtail squid (Sepiolidae: Sepiolinae) from the Ryukyu islands revealed by molecular and morphological analysis
Source: Commun Biol. 2019 Dec 11;2:465. doi: 10.1038/s42003-019-0661-6 (PMC6906322; doi:10.1038/s42003-019-0661-6)
Supplement: Supplementary file 1 — Supplementary Information [file 42003_2019_661_MOESM1_ESM.pdf]

SUPPLEMENTARY INFORMATION

Supplementary Figures:

Supplementary Figure 1. Pairwise synonymous distance (Ks) within species, within genera and between genera.
Supplementary Figure 2. Hatchlings of *Euprymna* sp. Type 1 and *Euprymna parva*.

Supplementary Tables:

Supplementary Table 1. Additional reference sequences used with their corresponding reference and GenBank accession numbers.

Supplementary Table 2. Mitochondrial cytochrome oxidase I sequence samples.

Supplementary Table 3. *Euprymna brenneri*, sp. nov.: measurements (mm), counts and indices of type specimens of both sexes.

Supplementary Table 4. Definitions of measurements and counts for morphological analysis.

Supplementary Notes:

Supplementary Note 1. COI sequencing reveals likely species misidentifications in public datasets.

Supplementary Note 2. The nature of Type I Ryukyu bobtail squid.

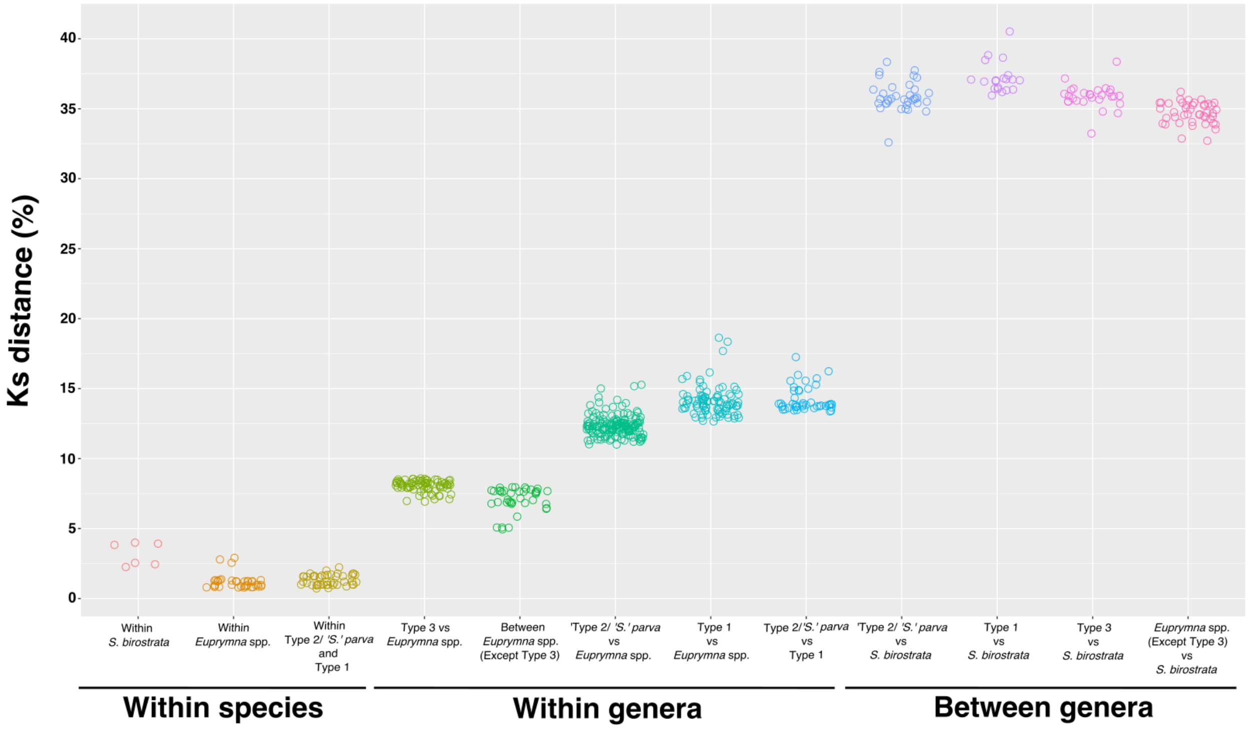

Supplementary Figure 1. Pairwise synonymous distance (Ks) within species, within genera and between genera.

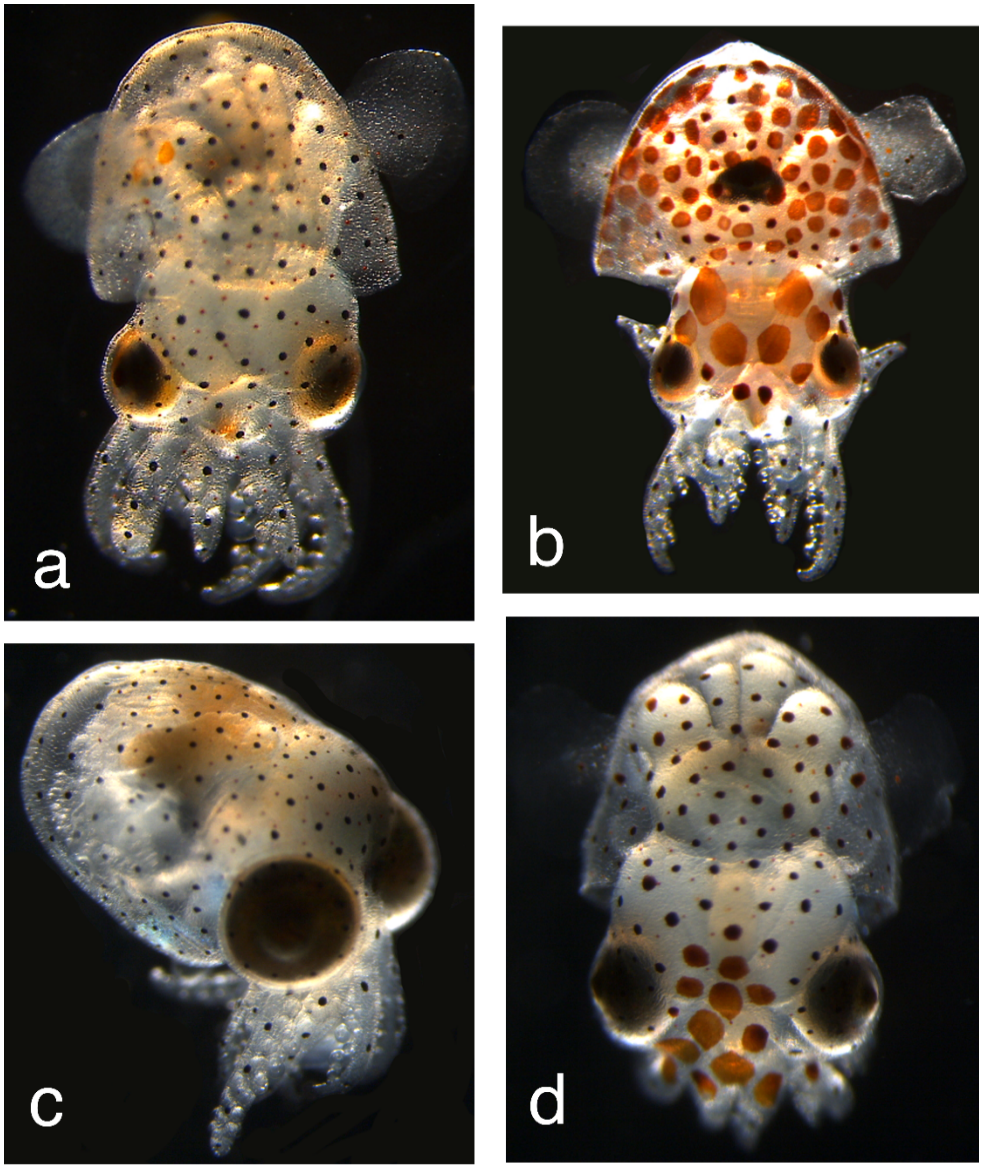

Supplementary Figure 2. *Euprymna* sp. Type 1 and *Euprymna parva* hatchlings. *Euprymna* sp. Type 1 hatchlings: **a** Dorsal view, with relaxed chromatophores; **b** with expanded chromatophores. *Euprymna parva* hatchlings: **c** dorso-lateral view, right; **d** dorsal view.

Supplementary Table 1. Additional reference sequences used with their corresponding reference and GenBank accession numbers. Suspected original misidentifications: *, likely *E. hyllebergi*; **, likely *E. morsei*; +, likely *S. birostrata.*

Samples	Genbank accession Number	Location	Reference
Sepiolina petasus Kubodera & Okutani, 2011	AB591071	Kumeshima Island	Kubodera and Okutani 2011
Euprymna albatrossae Voss, 1962 H38**	MF379400	Puerto Bay, Palawan, The Philippine archipelago	Coryell *et al.,* 2018
Euprymna albatrossae H39**	MF379401	Puerto Bay, Palawan, The Philippine archipelago	Coryell *et al.,* 2018
Euprymna albatrossae H2	MF379364	San Juan Barotac, Panay, The Philippine archipelago	Coryell *et al.,* 2018
Euprymna albatrossae H13	MF379375	The Philippine archipelago	Coryell *et al.,* 2018
Euprymna albatrossae H12	MF379374	The Philippine archipelago	Coryell *et al.,* 2018
Euprymna albatrossae H35	MF379397	The Philippine archipelago	Coryell *et al.,* 2018
Euprymna albatrossae H21	MF379383	Panay, The Philippine archipelago	Coryell *et al.,* 2018
Euprymna albatrossae H43	MF379405	The Philippine archipelago	Coryell *et al.,* 2018
Euprymna hyllebergi Nateewathana, 1997 H26	DQ646712	Rayong, Thailand	Jones *et al.*, 2006
Euprymna hyllebergi	AY293714	Gulf of Thailand	Nishiguchi *et al.*, 2004
Euprymna hyllebergi H27	DQ646711	Rayong, Thailand	Jones *et al.*, 2006
Euprymna berryi Sasaki, 1929 G08 – China*	HQ846101	Yangjiang, China	Dai *et al.*, 2012
Euprymna berryi G01 – China*	HQ846099	Beihai China	Dai *et al.*, 2012

Euprymna hyllebergi H28	DQ646712	Rayong, Thailand	Jones *et al.*, 2006
Euprymna berryi – Japan**	AY293711	Tosa Bay, Japan	Nishiguchi *et al.*, 2004
Euprymna morsei Verrill, 1881 H04 – China	HQ846105	Rizhao, China	Dai *et al.*, 2012
Euprymna morsei H02 – China	HQ846104	Rizhao, China	Dai *et al.*, 2012
Euprymna tasmanica Pfeffer, 1884 H37	DQ646729	Shark Bay, WA, Australia	Jones *et al.*, 2006
Euprymna tasmanica H16	DQ646727	Kurnell, Botany Bay, Sydney, NSW, Australia	Jones *et al.*, 2006
Euprymna tasmanica H36	DQ646728	Shark Bay, WA, Australia	Jones *et al.*, 2006
Euprymna tasmanica	AY293713	Melbourne, Australia	Nishiguchi *et al.*, 2004
Euprymna scolopes Berry, 1913	AY293713	Paiko, Honolulu	Nishiguchi *et al.*, 2004
Euprymna scolopes H4	DQ646739	Lilli'puna Pier, Kane'ohe Bay, Hawaii, USA	Jones *et al.* 2006
Euprymna scolopes H8	DQ646738	Coconut Island, Kane'ohe Bay, Hawaii, USA	Jones *et al.*, 2006
Sepiola pfefferi Grimpe, 1921	KM517947	North and Baltic Sea	Gebhardt and Knebelsberger 2015
Heteroteuthis dagamensis Robson, 1924	KR606071	Gulf of Mexico	Judkins *et al.*, 2016
Heteroteuthis hawaiiensis Berry, 1909	AY293728	Hokusei Maru, Japan	Nishiguchi *et al.*, 2004
Rossia pacifica Berry, 1911	GU802393	Haida Gwaii, British Columbia, Canada	Layton *et al.*, 2014
Rossia palpebrosa Owen, 1834	HM432253	Resolute Bay, Canada	Layton *et al.*, 2014
Rossia macrosoma Delle Chiaje, 1830	KM517936	North and Baltic Sea	Gebhardt and Knebelsberger 2015

Heteroteuthis ryukyuensis Kubodera, Okutani & Kosuge, 2009	AB591074	Near Okinawa, off Ishigaki Island, Japan	Kubodera *et al.*, 2009
Stoloteuthis japonica Kubodera & Okutani, 2011	AB591072	Okinawa, off Kumeshima Island, Japan	Kubodera and Okutani 2011
Sepiolina nipponensis Berry, 1911	AB591073	off Tosa Bay, Japan	Nishiguchi *et al.*, 2004
Sepiola affinis Naef, 1912	AY293716	Banyuls-sur-mer, France	Nishiguchi *et al.*, 2004
Heteroteuthis dispar Rüpell, 1844	AF035713	Atlantic Ocean	Nishiguchi *et al.*, 1998
Sepiola robusta Naef, 1912	AY293719	Banyuls-sur-mer, France	Nishiguchi *et al.*, 2004
Sepiola intermedia Naef, 1912	AY293718	Banyuls-sur-mer, France	Nishiguchi *et al.*, 2004
Sepiola ligulata Naef, 1912	AY293717	Banyuls-sur-mer, France	Nishiguchi *et al.*, 2004
Sepietta oweniana d'Orbugny [in Férussac & d'Orbigny], 1839–1842	KM517944	Banyuls-sur-mer, France	Nishiguchi *et al.*, 2004
Sepietta neglecta Naef, 1916	KM517940	North and Baltic Sea	Gebhardt and Knebelsberger 2015
Sepiola tridens de Heij & Goud, 2010	KM517963	North and Baltic Sea	Gebhardt and Knebelsberger 2015
Sepiola atlantica d'Orbugny [in Férussac & d'Orbigny], 1839–1842	KM517946	North and Baltic Sea	Gebhardt and Knebelsberger 2015
Sepiola rondeletii Leach, 1817	AY293720	Banyuls-sur-mer, France	Nishiguchi *et al.*, 2004
Sepietta obscura Naef, 1916	AY293723	Banyuls-sur-mer, France	Nishiguchi *et al.*, 2004
Sepiola birostrata Sasaki, 1918	AY293715	Tosa Bay, Kochi Prefecture, Japan	Nishiguchi *et al.*, 2004
Euprymna morsei +	AY293710	Tosa Bay, Kochi Prefecture, Japan	Nishiguchi *et al.*, 2004

Sepiadarium kochii Steenstrup, 1881	AB191292	Tosa Bay, Kochi Prefecture, Japan	Takumiya *et al.*, 2005

Supplementary Table 2. Mitochondrial cytochrome oxidase I sequence samples.

Sample / species	Haplotype	Genbank Accession Numbers	Ocean or Coastal Sea	Prefecture	Site	No. of Individuals
Type 3	Hap12*	LC417223	East China Sea	Okinawa	Seragaki	5
			Penghu water	–	Taiwan	1
			Pacific Ocean	Okinawa	Miyagi	1
			East China Sea	Okinawa	Kume	1
Type 3	Hap13	LC417224	East China Sea	Okinawa	Ishigaki	3
Type 3	Hap23**	LC417234	Pacific Ocean	Okinawa	Oura Bay	1
			Pacific Ocean	Okinawa	Miyagi	1
Type 2	Hap3***	LC417215	East China Sea	Okinawa	Seragaki	7
			East China Sea	Okinawa	Motobu	2
			Pacific Ocean	Okinawa	Miyagi	2
			East China Sea	Okinawa	Zamami	5
Type 2	Hap16	LC417227	East China Sea	Okinawa	Zamami	2
Type 2	Hap17	LC417228	East China Sea	Okinawa	Motobu	1
Type 1	Hap5	LC417217	East China Sea	Okinawa	Motobu	3
			East China Sea	Okinawa	Mizugama	1
Type 1	Hap4	LC417216	East China Sea	Okinawa	Motobu	1
Type 1	Hap6	LC417218	East China Sea	Okinawa	Ishigaki	3
Type 1	Hap11	LC417222	East China Sea	Okinawa	Mizugama	1
Euprymna berryi	Hap2	LC417214	Pacific Ocean	Mie	Unknown	2
Euprymna berryi	Hap19	LC417230	Seto Inland Sea	Yamaguchi	Mukoushima	1
Euprymna berryi	Hap22	LC417233	Penghu water	–	Taiwan	3
Euprymna berryi	Hap20	LC417231	Seto Inland Sea	Yamaguchi	Mukoushima	6
			Penghu water	–	Taiwan	2
Euprymna sp. (*morsei*)	Hap18	LC417229	Pacific Ocean	Mie	Unknown	4
Euprymna	Hap21	LC417232	Kaneohe Bay	–	Hawaii	5

scolopes						
Euprymna pardalota	Hap25	LC417212	Timor Sea	–	East Timor	1
Euprymna parva	Hap24	LC417211	Sagami Bay	–	Oshima	5
Sepiola birostrata	Hap9	LC417221	Seto Inland Sea	Hiroshima	Osaki - Shimozima	3
#*Sepiola birostrata*	Hap15	LC417226	Japan Sea	Hyogo	Unknown	4
Rossia sp.	Hap10	LC417235	Japan Sea	Hyogo	Unknown	1
†*Sepiadarium kochii*	Hap1	LC417213	Pacific Ocean	Mie	Unknown	2
†*Sepiadarium kochii*	Hap7	LC417219	Penghu water	–	Taiwan	1
†*Sepiadarium kochii*	Hap14	LC417225	Pacific Ocean	Okinawa	Miyagi	1
†*Sepioloidea lineolata*	Hap8	LC417220	–	–	Monterey Bay Aquarium	1

† Outgroup taxa from the family Sepiadariidae
* Haplotype for *E. brenneri* NSMT Mo 85892
** Haplotype for *E. brenneri* NSMT Mo 85891
*** Haplotype for *E. parva* AM C.574777
#*Euprymna parva* based on the re-description of *Sepiola parva* Sasaki 1913 in the main text

Supplementary Table 3. *Euprymna brenneri* sp. nov.: measurements (mm), counts and indices of type specimens of both sexes. –, values missing due to damage; n/a, not applicable.

**Museum Reg. no.**	**Paratype ♂ NMST Mo 85887**	**Paratype ♂ NMST Mo 85888**	**Holotype ♂ NMST Mo 85885**	**Paratype ♂ NMST Mo 85886**	**Paratype ♂ NMST Mo 85891**	**Paratype ♀ NMST Mo 85889**	**Paratype ♀ NMST Mo 85890**	**Paratype ♀ NMST Mo 85893**	**Paratype ♀ NMST Mo 85889**
ML	8.6	10.6	14.9	18.8	22.0	8.5	11.6	15.3	19.5
MWI	98.8	92.5	67.1	63.8	63.6	94.1	86.2	79.1	62.1
AMJI	79.1	63.2	53.7	47.9	45.5	70.6	54.3	58.8	48.7
VMLI	89.5	112.3	114.8	103.7	104.5	92.9	60.3	86.9	91.3
FWI	34.9	32.1	28.9	23.9	27.3	35.3	21.6	45.8	32.3
FIIa	27.9	26.4	26.8	31.9	31.8	32.9	25.9	26.1	25.6
FII	40.7	37.7	40.3	34.6	38.6	35.3	34.5	44.4	33.3
FuLI	52.3	75.5	67.1	65.4	55.5	67.2	67.2	65.4	52.3
FFuI	38.4	47.2	36.9	37.2	27.3	41.2	34.5	35.9	33.3
HLI	69.8	50.0	47.0	63.3	57.3	52.9	51.7	65.4	55.4
HWI	96.5	87.7	79.2	66.0	69.1	91.8	80.2	88.2	62.6
EDI	11.6	23.6	18.8	16.0	22.3	17.6	20.7	17.6	14.4
ALI1	46.5	80.2	94.0	–	–	49.4	53.4	55.6	61.5
ALI2	81.4	99.1	87.2	–	–	82.4	–	94.8	71.8
ALI3	89.5	94.3	97.3	–	–	74.1	71.6	81.7	92.3
ALI4	67.4	75.5	87.2	–	–	62.4	–	55.6	71.8
ASIn1	3.49	3.30	3.36	–	–	2.94	2.59	2.61	2.56
ASIn2	4.07	3.30	6.71	–	–	3.53	3.02	2.94	2.56
ASIn3	6.98	6.60	6.71	–	4.55	3.53	5.60	–	4.10
ASIn4	4.65	7.55	5.70	–	–	3.53	3.02	3.92	2.56
ASC1	70	76	72	–	–	84	90	80	74
ASC2	72	99	92	–	–	98	–	112	86
ASC3	70	94	64	–	–	65	88	78	84
ASC4	60	72	64	–	–	64	–	86	68
ClLI	18.6	33.0	20.1	–	22.7	32.9	23.3	29.4	17.9
ClRC	22	20	22	–	24	18	16	16	–
ClSI	0.58	0.38	0.27	–	0.36	–	0.43	0.33	0.31
GilLI	44.2	56.6	30.2	45.2	40.9	–	51.7	26.1	28.2
GiLC	27	26	27	27	25	–	25	24	24

EgDI	n/a	n/a	n/a	n/a	n/a	–	–	6.2	–
SpLI	–	–	–	–	54.5				–
SpWI	–	–	–	–	2.3				–

Supplementary Table 4. Definitions of measurements and counts for morphological analysis. Definitions largely follow Roper and Voss[79] and Reid[39]. Indices (shown in square brackets) are calculated by expressing each measure as a percentage of mantle length.

Anterior Mantle Join – **AMJ**: width of attachment of mantle to head [**AMJI**].

Arm Length – **AL**: length of each designated (i.e. 1, 2 etc.) arm measured from first basal (proximal-most) sucker to distal tip of arm (Arm 1, dorsal; 2, dorso-lateral; 3, ventro-lateral; 4, ventral) [**ALI**].

Arm Sucker Count **ASC**: total number of suckers on each designated arm (e.g. ASC2).

Arm Sucker diameter – **AS**: diameter of largest normal sucker on each designated (i.e. 1, 2 etc.) arm [**ASIn**].

Club Length – **ClL**: length of tentacular club measured from proximal-most basal suckers to distal tip of club [**ClLI**].

Club Row Count – **ClRC**: number of suckers in transverse rows on tentacular club.

Club Sucker diameter – **ClS**: diameter of largest sucker on tentacular club [**ClSI**].

Egg Diameter – **EgD**: diameter of largest egg present in the ovary or oviduct [**EgDI**].

Eye Diameter – **ED**: diameter of eye [**EDI**].

Fin Insertion – **FI**: length of fin as joined to mantle [**FII**].

Fin Insertion anterior – **FIa**: anterior origin of fin measured from mantle margin to anterior-most junction of fin and mantle [**FIIa**].

Fin Width – **FW**: greatest width of single fin [**FWI**].

Free Funnel length – **FFu**: the length of the funnel from the anterior funnel opening to the point of its dorsal attachment to the head [**FFuI**].

Funnel Length – **FuL**: the length of the funnel from the anterior funnel opening to the posterior margin measured along the ventral midline [**FuLI**].

Gill Length – **GiL**: length of the gill [**GiLI**].

Gill Lamellae Count – **GiLC**: number of lamellae in each demibranch.

Head Length – **HL**: dorsal length of head measured from point of fusion of dorsal arms to anterior tip of nuchal cartilage [**HLI**].

Head Width – **HW**: greatest width of head at level of eyes [**HWI**].

Mantle Length – **ML**: dorsal mantle length. Measured from anterior-most point of mantle to posterior apex of mantle.

Mantle Width – **MW**: greatest straight-line ventral width of mantle [**MWI**].

Spermatophore Length – **SpL**: length of spermatophore [**SpLI**].

Spermatophore Width – **SpW**: greatest width of spermatophore [**SpWI**].

Ventral Mantle Length – **VML**: length of ventral mantle measured along midline [**VMLI**].

Supplementary Note 1. COI sequencing reveals likely species misidentifications in public datasets
Our COI analysis revealed a few cases of public data in which *E. berryi*, *E. morsei*, *E. hyllebergi* Nateewathana, 1997, and *E. albatrossae* Voss, 1962 have apparently been misidentified, indicated by asterisks in Fig. 2. This could be due to the difficulty in obtaining mature males that are required for definitive identification. In order to resolve this confusion, we collected *E. berryi* from Mie Prefecture, Yamaguchi Prefecture, and Taiwan and verified their identification based on morphology. Ideally, definitive identifications should be based on material collected from the type locality of a species, but specimens from a number of localities in Japan were used when *E. berryi* was first described[1], so there is no single type locality. These samples group together as expected by the COI marker, forming a definitive *E. berryi* clade (Fig. 2). We also sequenced COI from one animal definitively verified as *E. morsei*, and a pair of *Sepiola birostrata*. Based on these new reference sequences from animals with morphologically confirmed species identities, we infer that the COI sequences reported as *E. berryi* by Dai *et al.*[2] from southeastern China, and by Nishiguchi *et al.*[3] from mainland Japan (Tosa Bay, Kochi Prefecture), were misidentified and were in fact *E. hyllebergi* and *E morsei*, respectively, based on their very close similarity with other COI sequences (Fig. 2). These alternative identifications are consistent with the known ranges of these species; in particular, the only two *Euprymna* species known in mainland Japan are *E. morsei* and *E. berryi* (and now *E. parva*. see below). An additional '*E. morsei'* sample from Nishiguchi *et al.*[36] groups with our *S. birostrata* sequences and previously deposited *S. birostrata* data, rather than *E. morsei*, and also appears to be misidentified. Finally, COI sequences from a recent study of bobtail squid from the Philippines deposited as *E. albatrossae*[4] form an apparently definitive *E. albatrossae* clade (Fig. 2) but some *E. albatrossae* haplotypes are also found mixed in with a putative *E. hyllebergi* group and one sequence is nested among representatives of an entirely different family, the Sepiadariidae. These latter '*E. albatrossae*' samples may be misidentified. Comparisons among voucher animals (if available) are needed for definitive resolution of *E. hyllebergi* and *E. albatrossae*. These findings underline the importance of retaining voucher animals from which tissue for sequencing is obtained.

Supplementary Note 2. Type 1 eggs were found near both Okinawa and the Ishigaki Islands and hatched in laboratory culture (Supplementary Fig. 2a, b). Type 1 hatchling transcriptomes are distinct from known adults of other bobtail squid distributed in the Ryukyus (Fig. 3).

We did not find wild adults corresponding to Type 1 eggs, and could not raise hatchlings to adults. Since diagnostic morphological characters for described species are known only in adults, we therefore could not formally identify Type I bobtails. Based on COI similarity, however, Type 1 appears to be closely related to *E. pardalota*, a species known from Australia and East Timor. Type I may be distinct from *E. pardalota* since the 5% COI divergence between them is somewhat larger than the 3.5%

COI divergence threshold used differentiate other closely related sepiolid species, e.g., between *Sepiolina nipponensis* Berry, 1911 and *Sepiolina petasus* Kubodera & Okutani, 2011 (subfamily Heteroteuthinae), and between *Sepiola tridens* de Heij & Goud, 2010 and *Sepiola atlantica* d'Orbigny 1842 (subfamily Sepiolinae)[5,6] (Although numerical divergence cutoffs should not be the only criteria to determine species boundaries because it assumes equivalent rates of evolutionary change among taxa.) Definitive analysis of Type I bobtails awaits the collection or culture of adult specimens, which should be compared with *E. pardalota* as well as *E. phenax* Voss 1962, described from the Phillippines, which Reid[7] reported was very similar morphologically to *E. pardalota* and may prove conspecific. Unfortunately, transcriptome data is not available for either *E. pardalota* or *E. phenax*.

REFERENCE

1. Pfeffer, G. Die Cephalopoden des Hamburger Naturhistorischen Museums. *Abhandlungen aus dem Gebiete der Naturwissenschaften / hrsg. vom Naturwissenschaftlichen Verein in Hamburg.* **8**, 1–30 (1885).
2. Dai, L., Zheng, X., Kong, L. & Li, Q. DNA barcoding analysis of Coleoidea (Mollusca: Cephalopoda) from Chinese waters. *Mol. Ecol. Resour.* **12**, 437–447 (2012).
3. Nishiguchi, M. K., Lopez, J. E. & Boletzky, S. V. Enlightenment of old ideas from new investigations: more questions regarding the evolution of bacteriogenic light organs in squids. *Evol. Dev.* **6**, 41–49 (2004).
4. Coryell, R. L., Turnham, K., de Jesus Ayson, E. G. & Lavilla-Pltogo, C. Phylogeographic patterns in the Philippine archipelago influence symbiont diversity in the bobtail squid–*Vibrio* mutualism. *Ecol. Evol.* **2018**, (2018).
5. Groenenberg, D. S. J., Goud, J., De Heij, A. & Gittenberger, E. Molecular phylogeny of North Sea Sepiolinae (Cephalopoda: Sepiolidae) reveals an overlooked Sepiola species. *J. Molluscan Stud.* **75**, 361–369 (2009).
6. De Heij, A. & Goud, J. *Sepiola tridens* spec. nov., an overlooked species (Cephalopoda, Sepiolidae) living in the North Sea and north-eastern Atlantic Ocean. *Basteria* **74**, 51–62 (2010).
7. Reid, A. *Euprymna pardalota* sp. nov. (Cephalopoda: Sepiolidae), a new dumpling squid from northern Australia. The Beagle, Records of the Museums and Art. *Galleries of the Northern Territory* **27**, 135–142 (2011).
