# Supplementary material for: New bobtail squid (Sepiolidae: Sepiolinae) from the Ryukyu islands revealed by molecular and morphological analysis
Source: Commun Biol. 2019 Dec 11;2:465. doi: 10.1038/s42003-019-0661-6 (PMC6906322; doi:10.1038/s42003-019-0661-6)
Supplement: Supplementary file 2 — Description of Additional Supplementary Items [file 42003_2019_661_MOESM2_ESM.pdf]

DESCRIPTION OF ADDITIONAL SUPPLEMENTARY ITEMS

Supplementary Data. Summary of transcriptome samples.
