# Supplementary material for: New bobtail squid (Sepiolidae: Sepiolinae) from the Ryukyu islands revealed by molecular and morphological analysis
Source: Commun Biol. 2019 Dec 11;2:465. doi: 10.1038/s42003-019-0661-6 (PMC6906322; doi:10.1038/s42003-019-0661-6)
Supplement: Supplementary file 4 — Reporting Summary [file 42003_2019_661_MOESM4_ESM.pdf]

nature research

Corresponding author(s): Daniel S. Rokhsar, Oleg Simakov

Last updated by author(s): Oct 4, 2019

Reporting Summary

Nature Research wishes to improve the reproducibility of the work that we publish. This form provides structure for consistency and transparency in reporting. For further information on Nature Research policies, see Authors & Referees and the Editorial Policy Checklist.

Statistics

For all statistical analyses, confirm that the following items are present in the figure legend, table legend, main text, or Methods section.

n/a	Confirmed	
☒	☐	The exact sample size (*n*) for each experimental group/condition, given as a discrete number and unit of measurement
☒	☐	A statement on whether measurements were taken from distinct samples or whether the same sample was measured repeatedly
☒	☐	The statistical test(s) used AND whether they are one- or two-sided<br>*Only common tests should be described solely by name; describe more complex techniques in the Methods section.*
☒	☐	A description of all covariates tested
☒	☐	A description of any assumptions or corrections, such as tests of normality and adjustment for multiple comparisons
☒	☐	A full description of the statistical parameters including central tendency (e.g. means) or other basic estimates (e.g. regression coefficient) AND variation (e.g. standard deviation) or associated estimates of uncertainty (e.g. confidence intervals)
☒	☐	For null hypothesis testing, the test statistic (e.g. *F*, *t*, *r*) with confidence intervals, effect sizes, degrees of freedom and *P* value noted<br>*Give P values as exact values whenever suitable.*
☐	☒	For Bayesian analysis, information on the choice of priors and Markov chain Monte Carlo settings
☒	☐	For hierarchical and complex designs, identification of the appropriate level for tests and full reporting of outcomes
☒	☐	Estimates of effect sizes (e.g. Cohen's *d*, Pearson's *r*), indicating how they were calculated

Our web collection on statistics for biologists contains articles on many of the points above.

Software and code

Policy information about availability of computer code

Data collection	NCBI
Data analysis	OMA stand-alone v.1.1.2, IQ-tree software 1.6.7, ModelFinder, Exabayes v1.5

For manuscripts utilizing custom algorithms or software that are central to the research but not yet described in published literature, software must be made available to editors/reviewers. We strongly encourage code deposition in a community repository (e.g. GitHub). See the Nature Research guidelines for submitting code & software for further information.

Data

Policy information about availability of data

All manuscripts must include a data availability statement. This statement should provide the following information, where applicable:

- Accession codes, unique identifiers, or web links for publicly available datasets
- A list of figures that have associated raw data
- A description of any restrictions on data availability

Study accession number for RNA sequencing: SRP157062. Accession number for COI sequences: LC417211–LC417235. Alignments, best partition scheme and models are available in Figshare (https://figshare.com/s/94a59f3ce82cacabaa7c).

Field-specific reporting

Please select the one below that is the best fit for your research. If you are not sure, read the appropriate sections before making your selection.

☐ Life sciences ☐ Behavioural & social sciences ☒ Ecological, evolutionary & environmental sciences

October 2018

Ecological, evolutionary & environmental sciences study design

All studies must disclose on these points even when the disclosure is negative.

Study description	Characterization of bobtail squid from the Ryukyu Island using DNA barcoding, transcriptome sequences, and morphology
Research sample	Optic lobe from squids' hatchlings and sometimes the mantle muscle of adults.
Sampling strategy	From each location, we randomly selected hatchlings from egg clutches collected in the wild. Adults were collected at night.
Data collection	Hatchlings and adults were anesthetized to dissect their optic lobe or muscle mantle.
Timing and spatial scale	February to November over several years, principally in the Ryukyu Islands.
Data exclusions	N/A
Reproducibility	N/A
Randomization	N/A
Blinding	N/A

Did the study involve field work? ☒ Yes ☐ No

Field work, collection and transport

Field conditions	Egg clutches and adult squids were collected at day and night time, respectively using SCUBA.
Location	The Ryukyu Island
Access and import/export	Bobtail squid are not categorized as a fish resource and presents no commercial value to fishermen. Regardless and whenever was needed, we contacted Onna's, Motobu's, and Yonashiro's fisherman Unions to request sampling at their area.
Disturbance	N/A

Reporting for specific materials, systems and methods

We require information from authors about some types of materials, experimental systems and methods used in many studies. Here, indicate whether each material, system or method listed is relevant to your study. If you are not sure if a list item applies to your research, read the appropriate section before selecting a response.

Materials & experimental systems

n/a	Involved in the study	
☒	☐	Antibodies
☒	☐	Eukaryotic cell lines
☒	☐	Palaeontology
☒	☐	Animals and other organisms
☒	☐	Human research participants
☒	☐	Clinical data

Methods

n/a	Involved in the study	
☒	☐	ChIP-seq
☒	☐	Flow cytometry
☒	☐	MRI-based neuroimaging
